# A Follow-Up Study After Reduction in an Excessive Intake of Cadmium from Rice on Changes in Blood and Urinary Cadmium Levels and Renal Tubular Function Among Female Farmers in Cadmium-Polluted Areas in Northern Japan

**DOI:** 10.3390/toxics14100872

**Published:** 2026-09-30

**Authors:** Hyogo Horiguchi, Etsuko Oguma, Kayoko Miyamotoa, Yoko Hosoi, Fujio Kayama

**Affiliations:** 1 Department of Hygiene, Kitasato University School of Medicine, Sagamihara 252-0374, Japan; oguma@med.kitasato-u.ac.jp; 2 Department of Environmental and Preventive Medicine, School of Medicine, Jichi Medical University, Shimotsuke-Shi 329-0498, Japan; kayokomy@nifty.com (K.M.); yk_hosoi2005@yahoo.co.jp (Y.H.); kayamafujio@gmail.com (F.K.)

**Keywords:** cadmium, rice, renal tubular function, farmers, follow-up study

## Abstract

There have been several cadmium-polluted areas in the vicinity of mines or smelters in Japan, where local inhabitants are at risk of overexposure to cadmium, which can induce renal tubular dysfunction. As part of the Japanese multi-centered environmental toxicant study (JMETS), we performed health examinations on female farmers in two adjacent cadmium (Cd)-polluted areas in Akita prefecture, northern Japan. Health examinations were conducted in area A in 2001–2002 and in area B in 2003–2004. The level of Cd pollution was higher in area B than in area A. After the initiation of the flooding of paddy fields to reduce rice Cd concentrations in 2002, we conducted a follow-up study in 2006 and 2008 on the same farmers in areas A and B, respectively. Among the 725 and 438 farmers who participated in the first health examinations, 534 and 356 participated in the follow-up, with follow-up rates of 73.7 and 81.3% in areas A and B, respectively. Of these, we selected 763 eligible female farmers aged 34–83 years in the follow-up for analysis. Cd concentrations in the farmers’ home-harvested rice markedly decreased, indicating a reduction in their Cd intake from rice. However, blood Cd levels did not change because the decrease in Cd intake may have been too small to affect high baseline blood Cd levels. On the other hand, urinary Cd levels increased and decreased from the lower and higher baseline levels of urinary Cd levels, respectively. Renal tubular function, indicated by urinary α1-microglobulin and ß2-microglobulin levels, remained unchanged; however, farmers ≥70 years with a baseline urinary Cd level ≥7 µg/g cr. had elevated urinary α_1_-microglobulin and ß_2_-microglobulin levels, indicating the deterioration of renal tubular function. Among them, 3 farmers had Cd nephropathy, presenting with rapid and marked increases in urinary ß_2_-microglobulin levels during the follow-up period. Collectively, these results provide a temporal picture of the sequential development of Cd nephropathy: an individual exposed to a high level of Cd for a long time develops renal tubular dysfunction gradually when older than 70 years, and Cd nephropathy then suddenly occurs and rapidly progresses.

## 1. Introduction

Cadmium (Cd) is a heavy metal that exists ubiquitously in the environment and is bio-concentrated in grains, vegetables, and marine products, through the consumption of which humans intake Cd. With a low absorption rate in the intestines (generally <10%) and a trace level of excretion (0.01–0.02%/day of total accumulated Cd), Cd accumulates in the kidneys after its absorption in a very slow and constantly increasing pattern from birth, resulting in the long biological half-life of Cd (approximately 10–30 years) [1]. In the kidneys, Cd exists in renal tubular cells by binding with metallothionein (MT) [2], and has no adverse effects at a low level of accumulation. However, the overaccumulation of Cd in the kidneys overcomes the protective effects of MT and induces the gradual deterioration of renal tubular function with aging.

The renal accumulation level of Cd is generally markedly less than the threshold to induce renal tubular dysfunction, which is approximately 200 mg/kg in the renal cortex [1]. However, inhabitants in local Cd-polluted areas, which in most cases are in the vicinity of mines or smelters, are at risk of overexposure to Cd. In Japan, there have been several Cd-polluted areas, with the most serious one being the Jinzu River basin in Toyama prefecture, located in the central part of the Japan Sea side [3]. Rice fields in the area were heavily polluted by Cd present in river water contaminated by waste from Kamioka mine. Many farmers had renal tubular dysfunction due to oral exposure to a high level of Cd through Cd-contaminated rice and river water. More than 200 farmers developed itai-itai disease, the most severe case of Cd toxicity that presented with osteomalacia following renal tubular dysfunction [4,5].

There were also large Cd-polluted areas in Akita prefecture, located in the northern part of Japan, which were equal in size to those in Toyama prefecture [6]. Many mines of various scales and their affiliated smelters were operated throughout Akita prefecture, resulting in a scattered distribution of Cd-polluted areas in the northern, central, and southern regions. There are large areas of rice fields in Akita Prefecture, and the fields near the mines and smelters were contaminated with Cd through the irrigation of polluted river water and contaminated air emissions. Consequently, rice produced in these fields contained excessive amounts of Cd, and local farmers were orally exposed to Cd through the lifelong consumption of home-harvested rice, leading to the accumulation of Cd in their bodies.

Kosaka town in the northern Cd-polluted region in Akita was intensively investigated in the 1970s to show that local farmers were excessively exposed to Cd and their renal tubular function was obviously affected [7,8]. Based on these findings, we conducted expanded investigations in the northern Cd-polluted region in 2001–2004, including Odate city and Kazuno city in addition to Kosaka town, as part of the Japanese multi-centered environmental toxicant study (JMETS), which examined the health effects of Cd exposure through the consumption of self-harvested rice in female farmers in areas of Japan with various levels of Cd pollution [9]. We demonstrated that the Cd exposure levels of female farmers were higher in Kosaka town and Kazuno city, where large mines with smelters existed, than in Odate city, located downstream of the mines and through which the Yoneshiro River flows, and those in their 70s had deteriorated renal tubular function.

The flooding of paddy fields in rice farming before and after heading during August, which is a very effective measure to lower Cd absorption by rice from the soil [10], was started in Cd-polluted areas throughout Akita prefecture in 2002. According to the Department of Agriculture, Forestry and Fisheries of Akita prefecture, the rates of rice with a Cd concentration above the safety standard in the northern Cd-polluted region markedly decreased from approximately 30% in 2001 to almost zero in 2004 [11]. Since Cd intake from rice and rice products accounted for approximately 50% of the total Cd intake among the farmers in the Cd-polluted region at that time [11], their Cd exposure level was also expected to have markedly decreased from the initiation of the flooding of paddy fields in 2002.

We herein conducted a follow-up investigation on farmers in Cd-polluted areas in the northern parts of Akita prefecture 4 to 5 years after the cross-sectional study in 2001–2004. Our aims were to confirm actual changes in Cd concentrations in home-harvested rice after the initiation of the flooding of paddy fields and also to examine changes in the levels of Cd that accumulated in the bodies of farmers and its effects on renal tubular function. This study may be regarded as a natural experiment on the effects of the abrupt reduction in excessive Cd exposure in individuals who had been highly and continuously exposed to Cd, which represents a rare opportunity that may never be possible again in the future.

## 2. Materials and Methods

### 2.1. Study Areas and Populations

The Committee on Medical Ethics of Jichi Medical University approved the research protocol, and this study was conducted in accordance with the ethical standards laid down in the Declaration of Helsinki. We previously investigated female farmers in their 20s-70s in Cd-polluted areas in the northern part of Akita prefecture that were separated into two areas based on the level of Cd pollution: area A in Odate city in November 2001–January 2002 and November 2002–December 2002, and area B in Kosaka town and Kazuno city in November 2003 and November 2004, obtaining 725 and 438 examinees, respectively. As noted above, the level of Cd pollution was higher in area B than in area A due to their geographical relationship with the mines, smelters and the river. Of the initial participants, 46 in Area A and 24 in Area B were lost to follow-up, which included 4 and 5 confirmed deaths, respectively. We reinvestigated areas A and B in 2006 and 2008, asking the remaining initial participants, 679 and 414, to participate the follow-up study, respectively. As a result, we obtained 534 and 356 follow-up participants from the first investigation, indicating follow-up rates of 73.7 and 81.3% in areas A and B, respectively (Supplementary Figure S1). These studies were conducted with local Japan Agricultural Cooperatives and municipalities. Among the follow-up participants, we excluded subjects who had less than a 10-year history of eating locally harvested rice (*n* = 71), had a history of smoking (*n* = 35), rheumatoid arthritis (*n* = 13), sarcoidosis (*n* = 1), and had no urine samples (*n* = 4) (there was some overlap), similar to our previous study [10]. Therefore, 778 subjects were included in analyses: 483 from area A and 295 from area B.

### 2.2. Procedures for Health Examinations

We performed health examinations on follow-up participants as described in our previous study [9]. Approximately one week before the health examination, we explained the purpose of the study and its protocol to the applicants and obtained their written informed consent in group settings. We also gave them a questionnaire on their place of residence, the origin of rice consumed, medical history, including diseases and therapies, and smoking habits, and asked them to bring it to the health examination, where it was checked by nurses. They were also asked to bring small amounts of their polished home-harvested rice. In the health examination, which was held in the morning, peripheral blood and second-urine samples were collected before breakfast, and rice samples were obtained. We also measured the participants’ weight.

### 2.3. Analyses of Blood and Urine Samples

Whole blood samples were taken in two separate tubes: for Cd measurements with heparin, and for biochemical measurements without anticoagulants to prepare serum samples by centrifugation. Urine samples were placed on ice immediately after collection and divided into three tubes: one drop (about 50 µL) of 20% sodium carbonate was added to prevent the destruction of ß_2_-microglobulin (ß2MG) due to low pH [12], one drop (about 50 µL) of 0.1 mol nitric acid was added to stabilize Cd, and no additives were used for other measurements. Urinary α_1_-microglobulin (α1MG) and ß2MG as well as serum and urinary creatinine (Cr) levels were measured using a latex agglutination method and the Jaffe reaction method, respectively. Mitsubishi Kagaku Bio-Clinical Laboratories, Inc. (Tokyo, Japan) conducted all biochemical measurements. The estimated glomerular filtration rate (eGFR) was calculated as an indicator of renal glomerular function using the following equation for Japanese females: eGFR (mL/min/1.73 m^2^) = 194 × serum Cr − 1.094 × age − 0.287 × 0.739 [13].

### 2.4. Measurement of Cd Concentrations in Whole Blood, Urine, and Rice

Cd concentrations in whole blood, urine, and rice were measured using the same protocol at the baseline and in the follow-up, as previously described [9]. Briefly, whole blood and rice samples were decomposed by microwaving with nitric acid, and Cd concentrations were measured using HP4500 ICP-MS (Yokogawa Analytical Systems, Tokyo, Japan). Urine samples were mixed with nitric acid, held for 24 h, and Cd concentrations were then measured using flameless atomic absorption spectrometry (SIMAA 6000; Perkin Elmer, Norwalk, CT, USA). Ultrapur nitric acid (Kanto Kagaku, Tokyo, Japan) and nitric acid for the Ultratrace Analysis (Wako Pure Chemical Industries, Osaka, Japan) were used for these measurements. Indium and thallium were added to all samples as internal standards. The standard solutions for Cd, indium, and thallium were purchased from Wako Pure Chemical Industries. The effects of the sample matrix on Cd measurements were examined using standards, including CRM195 (Institute for Reference Materials and Measurements) for peripheral blood, 69,071 Level 1 (human urine) (Bio Rad, Hercules, CA, USA) for urine, and NIES No. 10 (the National Institute of Environmental Science in Japan) for rice. The results obtained at the baseline and in the follow-up for the standard substances were within reference ranges. The limit of quantitation (LOQ) was calculated at 10× the standard deviation (SD) of reagent blank measurements (*n* = 5). IDEA Consultants, Inc. (Metocean Environment Inc., Shizuoka, Japan) conducted the measurements of Cd concentrations.

### 2.5. Statistical Analysis

Data that followed a normal distribution, such as age and eGFR, were presented as arithmetic means with SDs, while others (Cd levels in rice, blood, and urine, and urinary α1MG and β2MG levels) were presented as medians with 25th and 75th percentiles. Before the analysis, values less than the LOQ or the minimal measurable limit were replaced with half the value. Normal probability plots were used in judgements on the normal distribution of these data. The concentrations of urinary substances were adjusted by urinary Cr concentrations and presented as “µg/g cr.” In comparisons of values between areas, including baseline and follow-up data, as well as among groups classified by urinary Cd and age, the Tukey–Kramer test or Steel–Dwass test was used as a parametric or non-parametric multiple comparison, respectively. The χ^2^ test was used to assess differences in distribution. Changes in groups classified by age were examined by the Jonckheere–Terpstra trend test. A test for differences in regression coefficients was applied for the comparison of regression equations. Statistical analyses were conducted using SPSS release 27.0 (SPSS Japan, Tokyo, Japan) based on the basic management of data by Mac Excel Tokei ver. 3.0 (Esumi, Tokyo, Japan).

## 3. Results

### 3.1. Cd Concentrations in Rice

**Figure 1:**
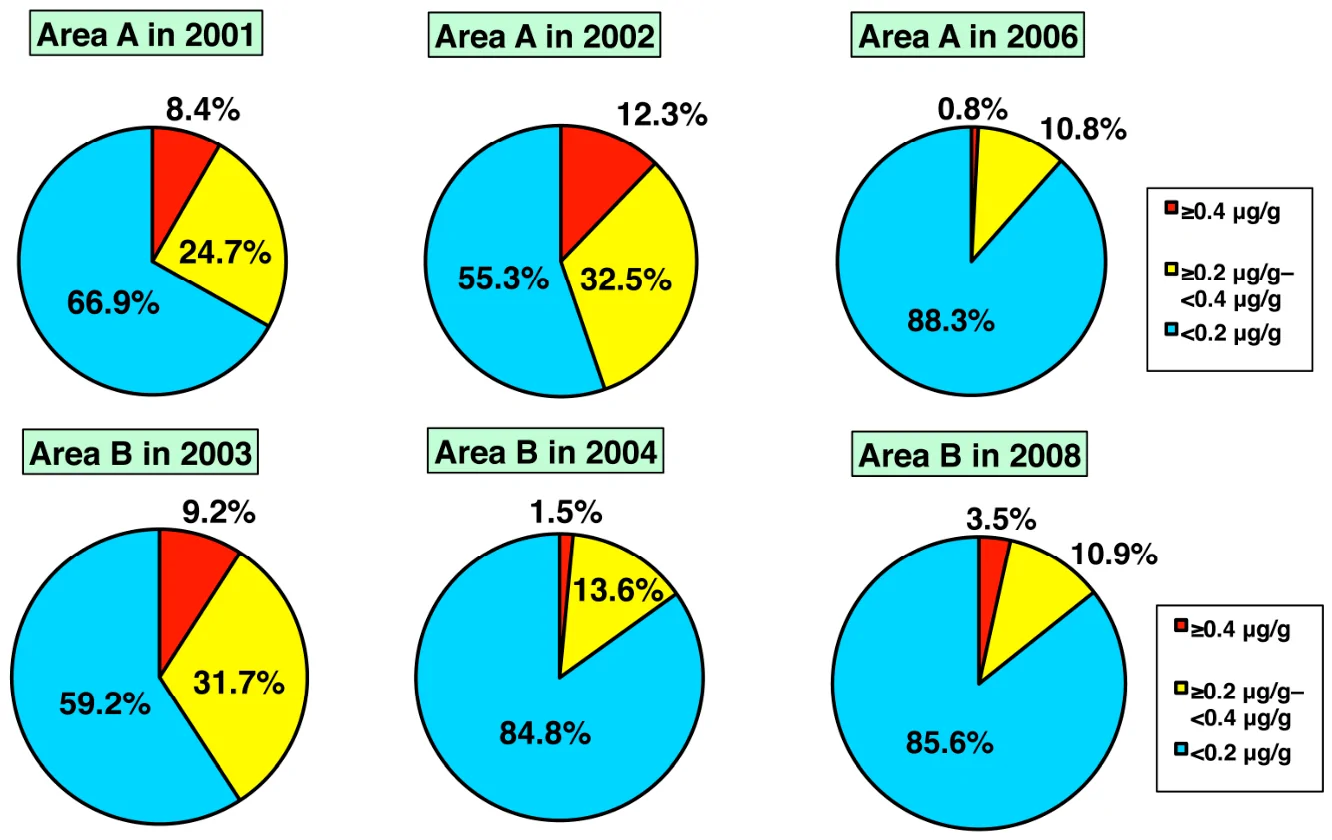
Chronological changes in the distribution of cadmium concentrations in rice (cut-offs were 0.2 and 0.4 μg/g) in areas A and B (*p* < 0.05 using the χ^2^ test in each area). Totals may not add up to 100% due to rounding.

We confirmed changes in Cd concentrations in rice before and after implementing the flooding of paddy fields, around 2002, in areas A and B (Table 1). The results obtained for area A in 2001 and 2002 and for area B in 2003 and 2004 were cited from our previous study [9]. In area A, the median rice Cd concentration was significantly lower in 2006 than in 2001 and 2002, just before the start of the flooding of paddy fields. On the other hand, in area B, median rice Cd concentrations decreased from 2003 to 2004, just after the start of the flooding of paddy fields, and remained low in 2008. The rates of rice with a Cd concentration above the safety standard (0.4 µg/g) were consistent with the medians: approximately 8–12% in 2001–2002 in area A, with a decrease to 0.8% in 2006, and approximately 9% in 2003 in area B, with a decrease to 1.5% in 2004 and 3.5% in 2008 (Figure 1). These results indicate that Cd concentrations in rice in areas A and B simultaneously started to decrease in 2003 and then reached a low plateau in 2004, suggesting a consequential decrease in farmers’ Cd intake.

### 3.2. Age Classification of Subjects for Analysis

Since the level of Cd that accumulated in the body and its renal effects are significantly affected by age, we classified subjects by their baseline age to analyze them without age effects (Table 2). In all age classes, mean ages at baseline and during the follow-up did not significantly differ between areas A and B. Differences in age between the baseline and follow-up were approximately 4.5–5 years. Since there were no subjects younger than 34 years in area B, 15 subjects younger than 34 years in area A were omitted from subsequent comparative analyses of the 2 areas (total number, 763).

### 3.3. Changes in Cd Levels in Peripheral Blood and Urine

We measured Cd levels in the peripheral blood and urine of subjects to assess actual changes in Cd accumulation in their bodies.

**Figure 2:**
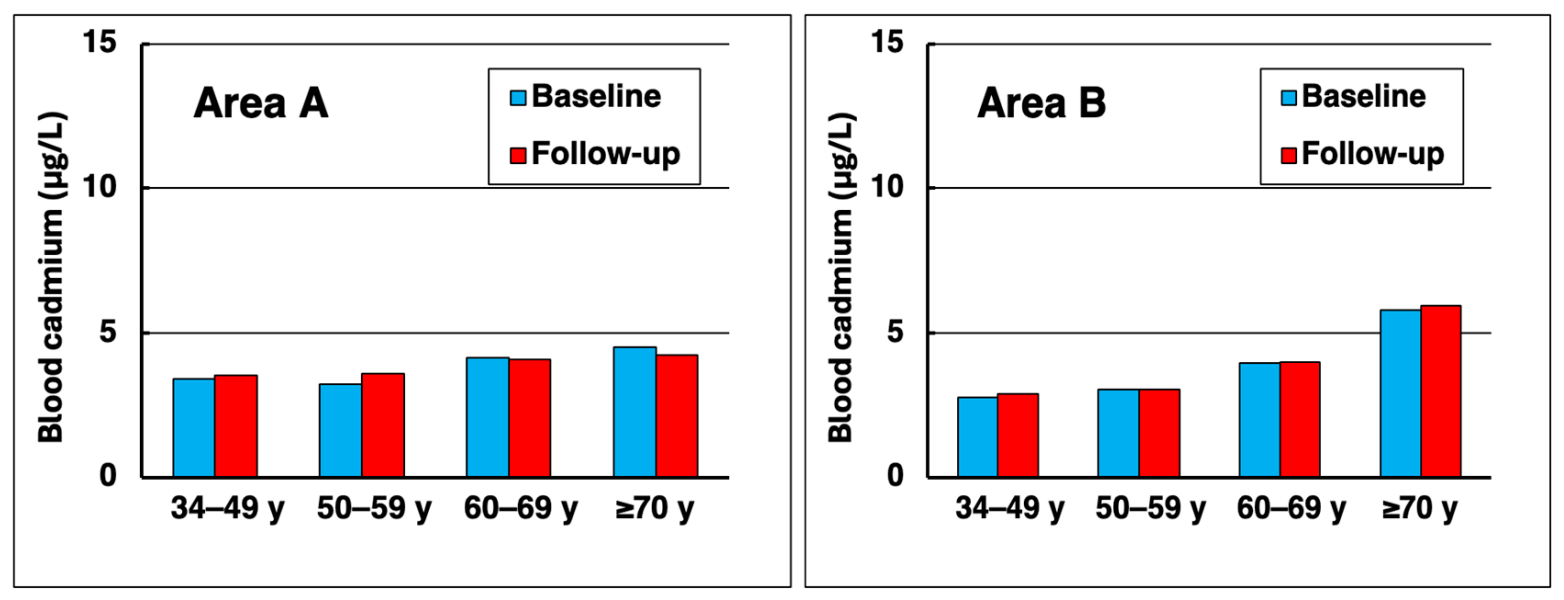
Changes in cadmium levels in peripheral blood during the follow-up period in age-classified subjects in areas A and B. Baseline and follow-up indicate the investigation years: 2001–2002 and 2006 in area A, and 2003–2004 and 2008 in area B, respectively.

Blood Cd levels were slightly lower in subjects in area B than in those in area A, and no significant changes were observed from the baseline to the follow-up in both areas (Table 3). The age classification showed that blood Cd levels increased with age in both areas, whereas those in the age classes of 34–49 and 50–59 years were slightly lower in area B than in area A, and those in the age class of ≥70 years were higher in area B than in area A at both the baseline and in the follow-up. On the other hand, no significant differences were observed in blood Cd levels between the baseline and follow-up in all age classes in both areas (Table 3, Figure 2).

**Figure 3:**
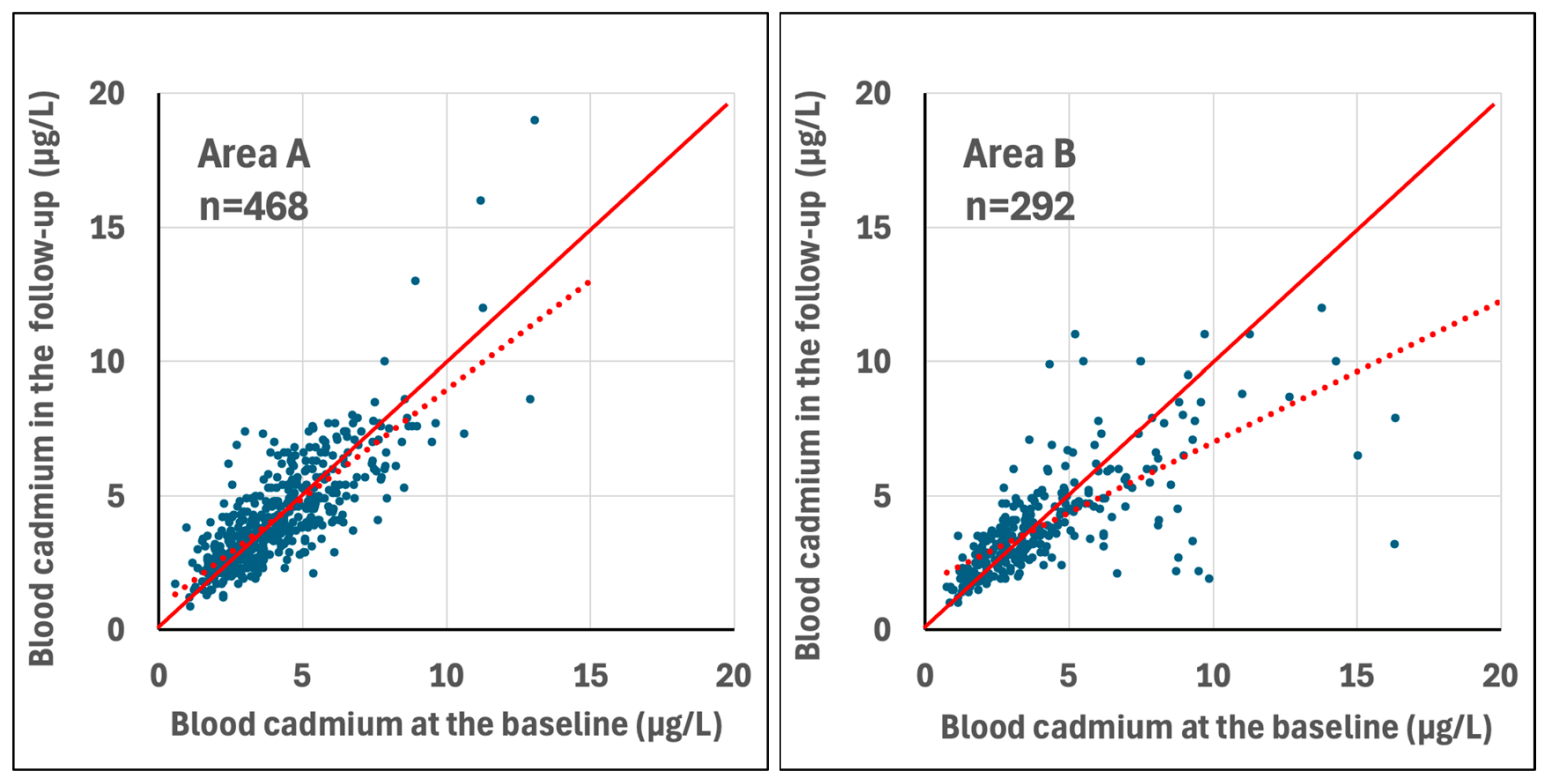
Relationships of blood cadmium concentrations at baseline and in the follow-up in areas A and B. Straight lines indicate hypothetical relationships with equal values for the baseline and follow-up, and dotted lines indicate actual linear regression lines between the baseline and follow-up.

Although overall changes in blood Cd levels during the follow-up period were not large in area A, blood Cd levels in subjects with markedly elevated baseline blood Cd levels markedly decreased in the follow-up in area B (Figure 3). Regression equations of the logarithmic values of blood Cd at the baseline and in the follow-up in areas A and B were y = 0.722x + 0.172 and y = 0.616x + 0.211, respectively, and the difference in the slopes was significant (*p* = 0.007). These changes were more clearly observed in age-classified subjects in areas A and B, particularly in older age classes in area B (Supplementary Figure S2). These results indicate that while blood Cd levels were maintained during the follow-up period, those in elderly subjects with higher blood Cd levels generally decreased.

**Figure 4:**
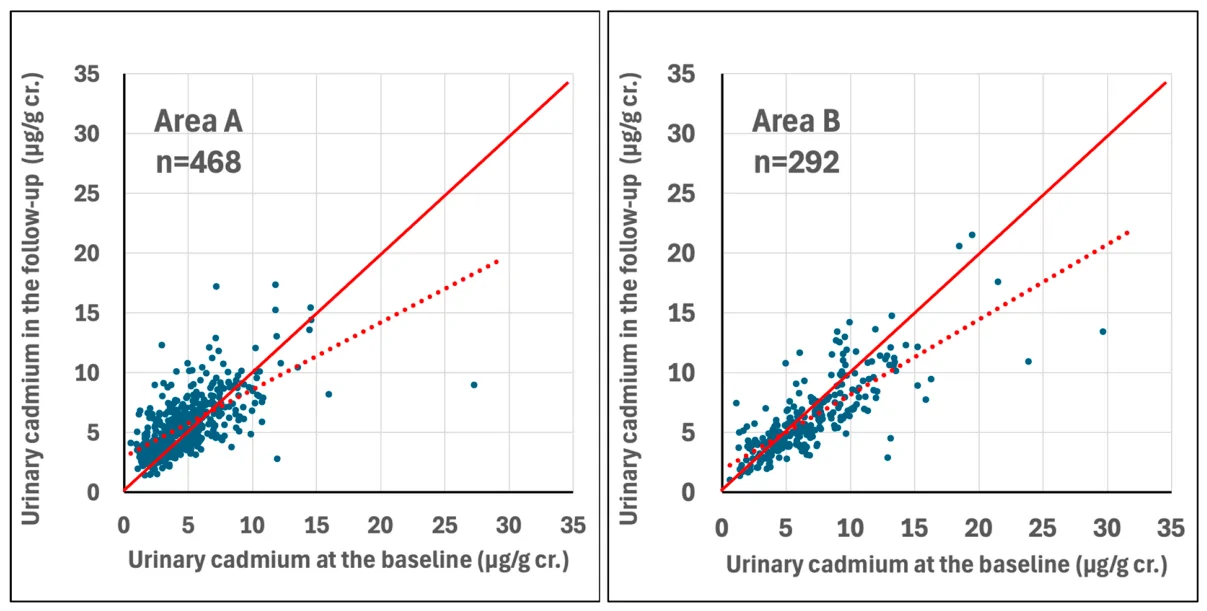
Relationships of urinary cadmium concentrations between the baseline and follow-up in each age class. Baseline and follow-up indicate the investigation years: 2001–2002 and 2006 in area A, and 2003–2004 and 2008 in area B, respectively. Straight lines indicate hypothetical relationships with equal values at the baseline and in the follow-up, and dotted lines indicate actual linear regression lines between the baseline and follow-up.

**Figure 5:**
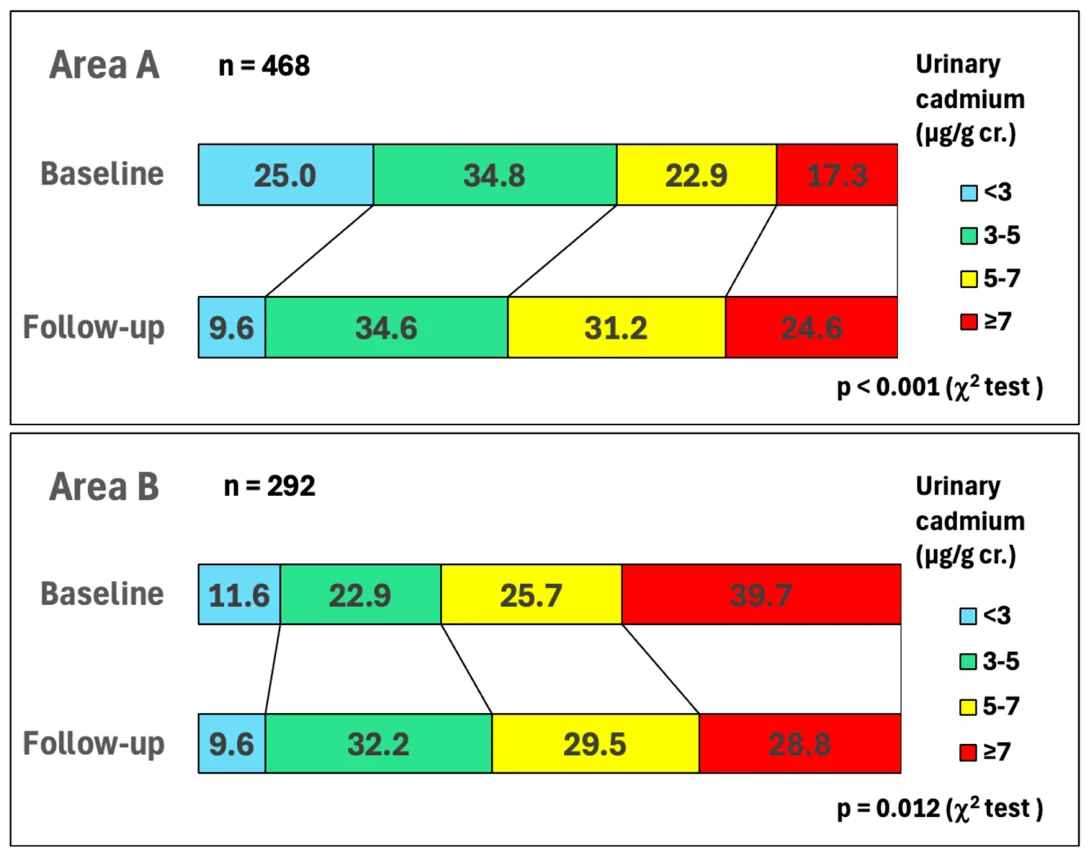
Changes in distributions of urinary cadmium levels between the baseline and follow-up in areas A and B. Baseline and follow-up indicate the investigation years: 2001–2002 and 2006 in area A, and 2003–2004 and 2008 in area B, respectively. Subjects of all ages were divided by urinary cadmium levels of 3, 5, and ≥7 µg/g cr., and data are presented as percentages.

On the other hand, urinary Cd levels were higher in subjects in area B at all ages than in those in area A at the baseline, whereas those in areas A and B increased and decreased from the baseline to the follow-up, respectively, resulting in similar levels in the follow-up (Table 4). Individual analyses of the relationships of urinary Cd levels at the baseline and in the follow-up show these changes in more detail (Figure 4). There were more subjects above the equal value line at lower baseline urinary Cd levels in area A and more subjects under the equal value line at higher baseline urinary Cd levels in area B. The distributions of urinary Cd levels showed similar patterns when stratified using cut off values of 3, 5, and 7 µg/g cr., which corresponded approximately to the 25th percentile (3.28 μg/g cr.), median (4.91 μg/g cr.), and 75th percentile (7.10 μg/g cr.) in 760 subjects, respectively,: the number of subjects with lower urinary Cd levels decreased and those with higher urinary Cd levels increased in area A, while the number of those with higher urinary Cd levels decreased in area B from the baseline to the follow-up (Figure 5).

**Figure 6:**
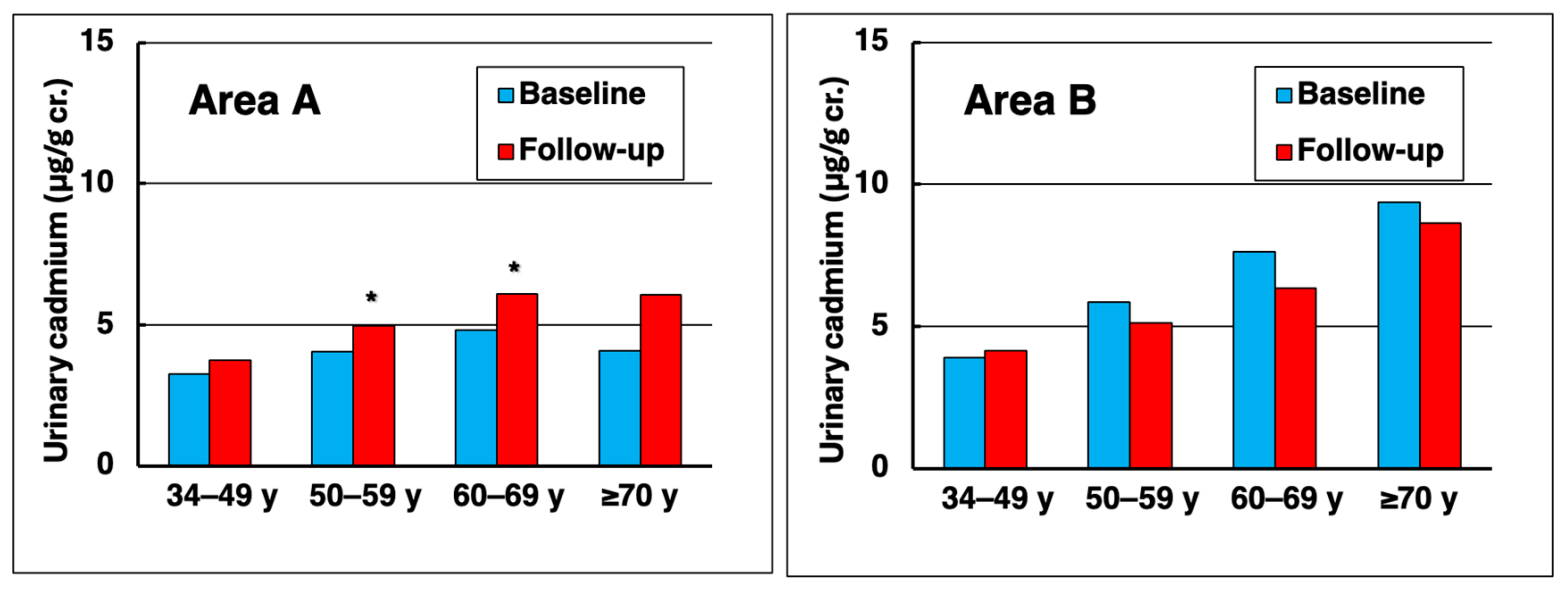
Changes in urinary cadmium levels during the follow-up period in age-classified subjects in areas A and B. Baseline and follow-up indicate the investigation years: 2001–2002 and 2006 in area A, and 2003–2004 and 2008 in area B, respectively. * Significant difference from the baseline (*p* < 0.05 using the Steel–Dwass test).

In age-classified observations of baseline urinary Cd levels, no significant differences were noted between age classes in area A, whereas an age-dependent increase was found in area B, which was more than two-fold higher in subjects ≥70 years than in those aged 34–49 years (Table 4, Figure 6). In contrast, in the follow-up, urinary Cd levels in area A significantly increased in the older age classes, while those in area B slightly decreased in the older age classes. Since the adjustment of urinary concentrations for urinary Cr is affected by skeletal muscles [14], overadjustment may have contributed to the observed age-dependent increase observed due to decreases in body weight with age. In fact, both urinary Cr and body weight showed age-dependent decreasing trends (Supplementary Table S1). However, their slopes were similar across all baseline and follow-up subgroups in both areas, whereas those for urinary Cd varied considerably among the subgroups. Therefore, the observed differences in the age-dependent trends of urinary Cd across the subgroups cannot be attributed to the adjustment for urinary Cr. These results indicate that older subjects with low urinary Cd levels showed an increase in urinary Cd, while those with high urinary Cd levels showed a decrease.

### 3.4. Urinary α1MG and ß2MG Levels and eGFR

**Figure 7:**
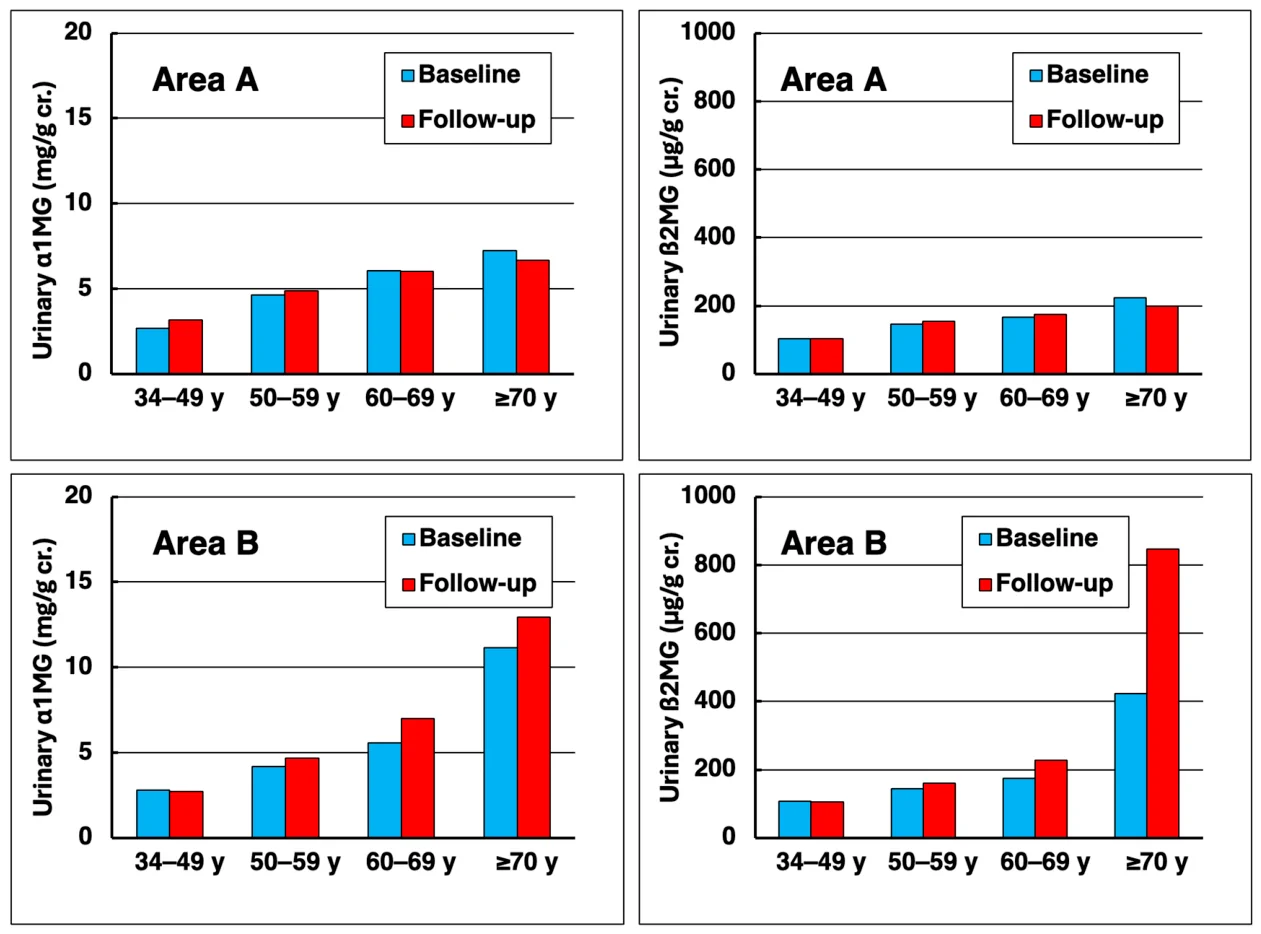
Changes in urinary α_1_-microglobulin (α1MG) and β_2_-microglobulin (β2MG) levels during the follow-up period in age-classified subjects in areas A and B. Baseline and follow-up indicate the investigation years: 2001–2002 and 2006 in area A, and 2003–2004 and 2008 in area B, respectively.

We measured changes in urinary α1MG and ß2MG concentrations, which are indicators of Cd-induced renal tubular dysfunction, in age-classified subjects (Table 5, Figure 7). Urinary α1MG and ß2MG levels in both areas showed age-dependent increases at both baseline and at follow-up, which may have been over-adjusted by urinary Cr concentrations that reflected age-dependent decreases in body weight (Supplementary Table S1). Nevertheless, changes were more pronounced in area B than in area A, and also in the follow-up than at the baseline in area B, with urinary ß2MG levels in subjects ≥70 years in the follow-up in area B being 4-fold higher than those in area A, and two-fold higher than those at the baseline. On the other hand, eGFR, which indicates renal glomerular function, also showed age-dependent decreases in both areas. While eGFR was lower in the follow-up than at the baseline in area A in each age class, it remained unchanged in the follow-up period in area B.

### 3.5. Net Relationship of Urinary Cd Levels and Renal Tubular Function

Based on the results of comparison between the 2 areas with different Cd exposure levels, we observed the net relationship of urinary Cd levels and renal tubular function, dividing the subjects by urinary Cd levels of 3, 5, and ≥7 μg/g cr., along with the age classification (Supplementary Table S2). We restricted the primary analysis to area A because the number of subjects under 70 years with a urinary Cd level < 7 µg/g cr. in area B was too small. Consequently, for area B, comparisons between age classes were performed exclusively within the subgroup of subjects with a urinary Cd level < 7 µg/g cr. (Supplementary Table S3).

**Figure 8:**
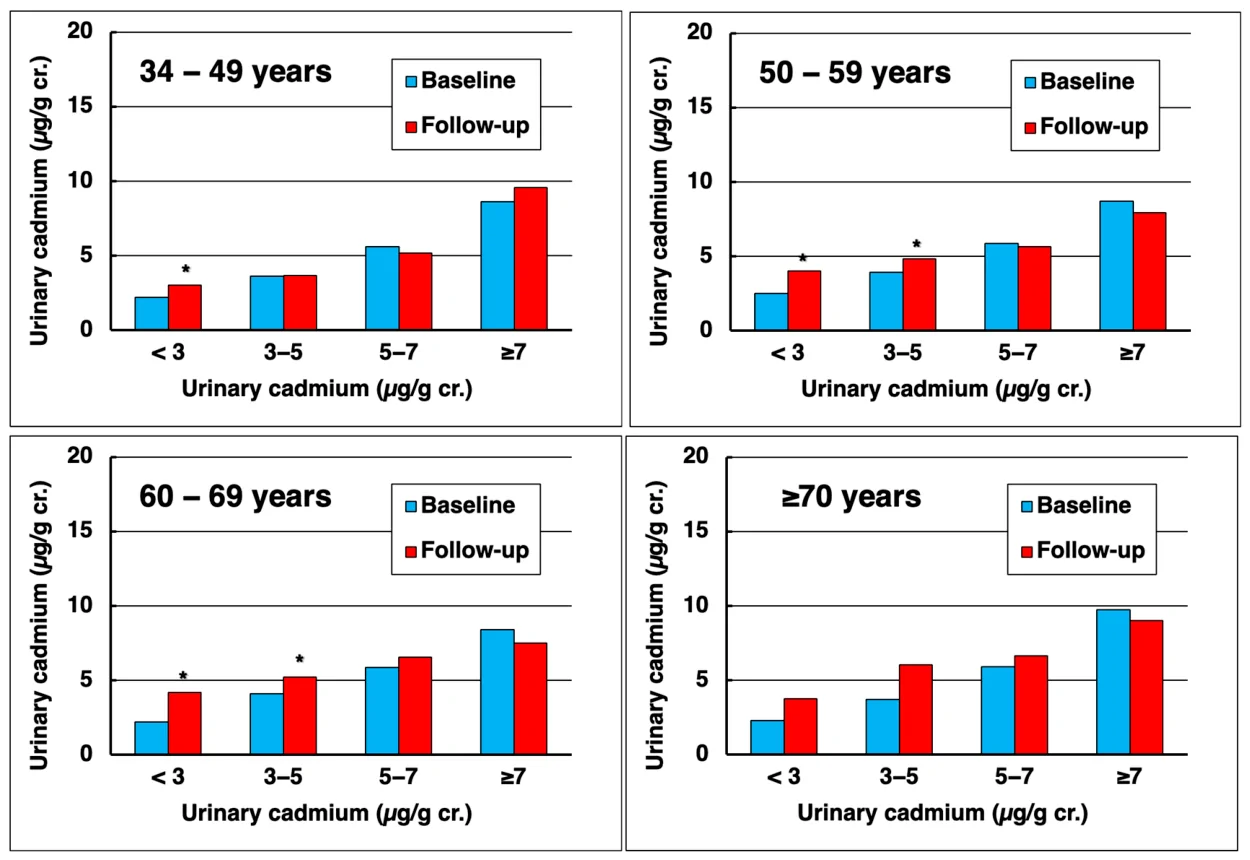
Changes in urinary Cd levels from the baseline to the follow-up at each baseline urinary Cd level in age-classified subjects in area A. Baseline and follow-up indicate the investigation years of 2001–2002 and 2006, respectively. * Significant difference from the baseline (*p* < 0.05 using the Steel–Dwass test).

**Figure 9:**
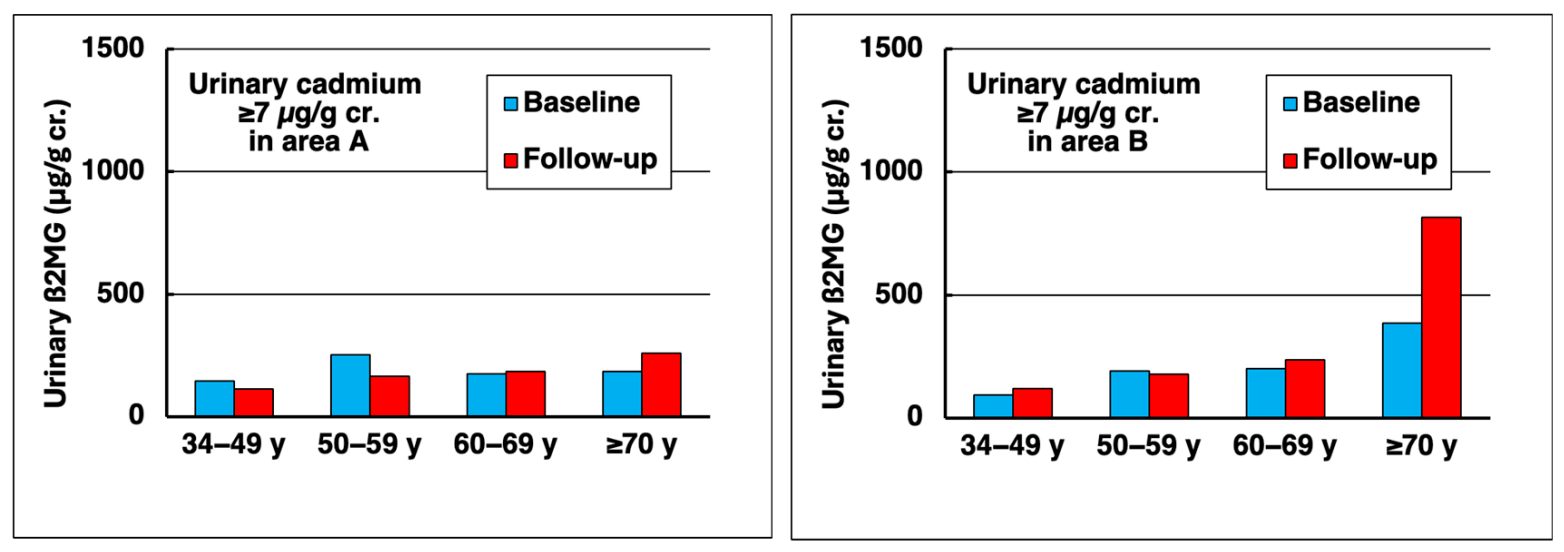
Changes in urinary β_2_-microglobulin (β2MG) levels from the baseline to the follow-up in age-classified subjects with a urinary Cd level <7 µg/g cr. from areas A and B. Baseline and follow-up indicate the investigation years: 2001–2002 and 2006 in area A, and 2003–2004 and 2008 in area B, respectively.

Blood Cd levels did not significantly change during the follow-up period in each age-classified and urinary Cd-divided subject group. In contrast, in all age classes during the follow–up period, urinary Cd levels generally increased in subjects with urinary Cd levels <~5 μg/g cr. at baseline and decreased in those with urinary Cd levels >~5 μg/g cr. at baseline (Figure 8). On the other hand, urinary α1MG and ß2MG levels did not change during the follow–up period at any urinary Cd level in subjects < 70 years in area A, whereas the urinary ß2MG level increased in those ≥70 years with a baseline urinary Cd level ≥ 7 μg/g cr. in area B (Figure 9).

### 3.6. Individual Analyses of Changes in Urinary ß2MG Level

**Figure 10:**
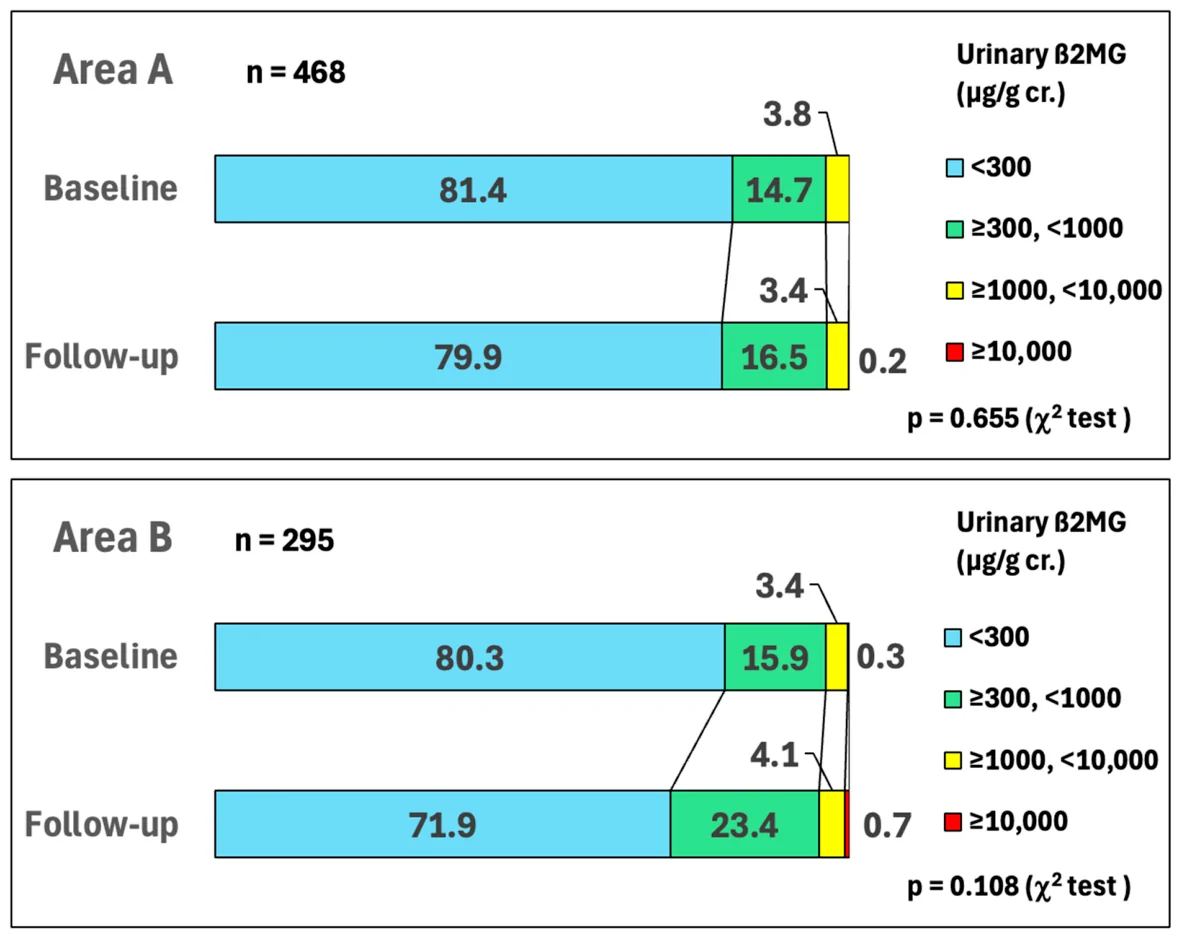
Changes in distributions of urinary ß_2_-microglobulin (ß2MG) levels between the baseline and follow-up in areas A and B. Subjects of all ages were divided by urinary ß2MG levels of 300, 1000, and 10,000 μg/g cr., and data are presented as percentages.

A few subjects had markedly elevated urinary ß2MG levels and were suspected to have Cd nephropathy (Table 5). Therefore, we divided subjects in areas A and B by urinary ß2MG levels of 300, 1000, and 10,000 μg/g cr., which are generally used as cut-off values for “incipient Cd tubulopathy”, “irreversible proteinuria”, and “overt Cd nephropathy”, respectively [15], to confirm changes in subjects affected by Cd (Figure 10). The distributions of urinary ß2MG levels were similar between the baseline and follow-up in area A. However, one subject with a urinary ß2MG level >10,000 μg/g cr. newly emerged during the follow-up period (0.2%), while all subjects had urinary ß2MG levels <10,000 μg/g cr. at baseline. On the other hand, in area B, the number of subjects with urinary ß2MG levels >300 μg/g cr. slightly increased during the follow-up period, and, more importantly, the number of subjects with urinary ß2MG levels >10,000 μg/g cr. increased from one (0.3%) to two (0.7%). Therefore, 3 subjects were suspected to have Cd nephropathy in the follow-up, with 2 newly developing this disease.

Among the subjects with a urinary β2MG level >10,000 μg/g cr., the two in area B exhibited very high blood and urinary Cd levels with no other renal diseases, whereas the subject in area A did not show elevated Cd levels. Therefore, the two subjects in area B were diagnosed with Cd nephropathy.

**Figure 11:**
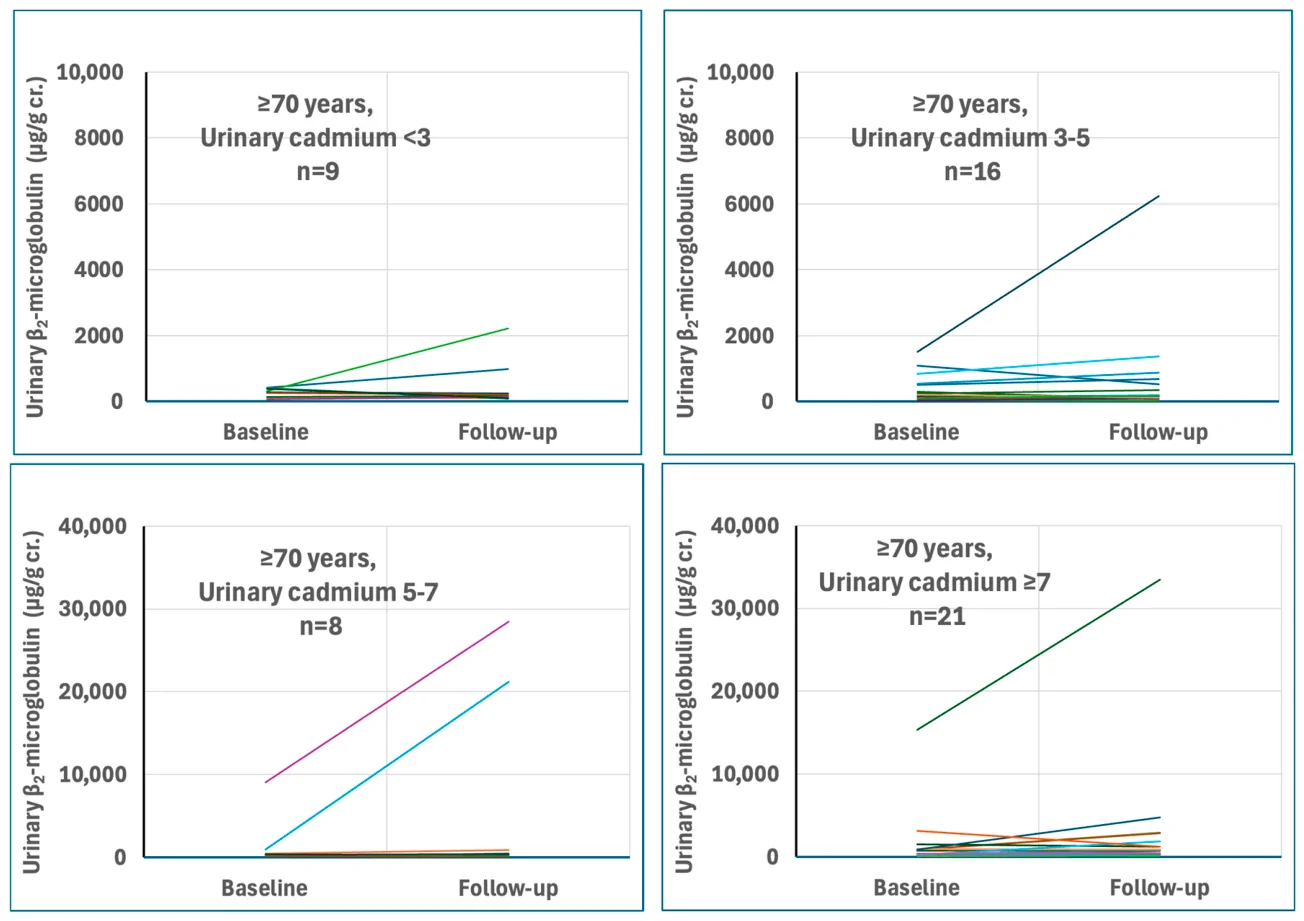
Individual changes in urinary ß_2_-microglobulin levels in subjects ≥ 70 years during the follow-up period, presented separately in groups divided by urinary cadmium levels of 3, 5, and ≥7 µg/g cr. Note the differences in the scales of urinary ß_2_-microglobulin levels. Different colors indicate different individuals.

To investigate the relevance of age and urinary Cd levels on changes in urinary ß2MG levels during the follow-up period in subjects, particularly those suspected to have Cd nephropathy, we examined individual changes in urinary ß2MG levels in integrated subjects by dividing by age and urinary Cd levels. Subjects <70 years in all urinary Cd-divided groups did not exhibit obvious individual changes in urinary ß2MG levels (Supplementary Figure S3A–C). Urinary ß2MG levels remained unchanged in the majority of subjects ≥ 70 years, except in 2 subjects in the 5–7 µg/g cr. group, levels rapidly increased from <10,000 µg/g cr. to more than 20,000 µg/g cr., and that in one subject in the ≥7 µg/g cr. group with a urinary ß2MG level of approximately 15,000 µg/g cr. at baseline showed a further increase to >30,000 µg/g cr. in the follow-up (Figure 11).

### 3.7. Follow-Up of One Subject After the Health Examination

**Figure 12:**
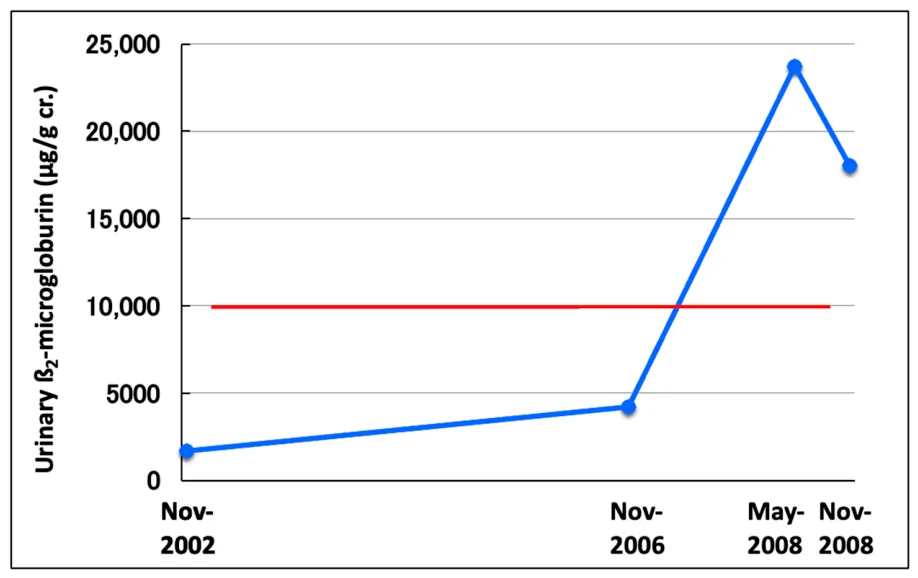
Chronological changes in urinary ß_2_-microglobulin levels in a subject who was followed up after the health examination in area A. The red line indicates a urinary ß_2_-microglobulin level of 10,000 µg/g cr.

One subject was followed up after the health examination to investigate the rapid progression of renal tubular dysfunction (Table 6, Figure 12). She participated in the health examination in area A in November 2002 at 66 years old, and showed high blood and urinary Cd levels without an elevation in the urinary ß2MG level. After 4 years, she participated in the follow-up health examination in November 2006 at 70 years old, and her urinary ß2MG level had slightly increased. After the follow-up health examination, she was followed up as one of the subjects with high blood or urinary Cd levels in May 2008. An abrupt elevation in her urinary ß2MG level to >23,000 µg/g cr. was noted when she was 71 years old. She participated in the follow-up health examination in area B in November 2008 at 72 years, and her urinary ß2MG level continued to be high. Because she had persistently elevated blood and urinary Cd levels without other renal diseases, she was diagnosed with Cd nephropathy.

## 4. Discussion

In this follow-up study on female farmers in two Cd-polluted areas in northern Akita prefecture for 4 to 5 years, we found a marked reduction in Cd concentrations in home-harvested rice after the start of the flooding of paddy fields, indicating that Cd intake from rice also decreased. However, blood Cd levels did not change, and urinary Cd levels increased and decreased from the lower and higher baseline urinary Cd levels, respectively. On the other hand, renal tubular function remained unchanged, while only elderly female farmers with higher baseline urinary Cd levels developed renal tubular dysfunction.

In area A, the rates of rice with a Cd concentration above the safety standard markedly decreased from approximately 10% in 2001–2002 to less than 1% in 2006, along with reductions in median rice Cd concentrations. This change was caused by the flooding of paddy fields that started in 2002. However, due to missing data on rice Cd concentrations in area B before 2003, actual changes in rice Cd concentrations derived from the flooding of paddy fields remain unclear. Nevertheless, the rates of rice with a Cd concentration above the safety standard decreased from approximately 9% in 2003 to 1.5% in 2004, which was considered to be in the middle of the downward trend from a markedly higher percentage. According to publicly available data from the Akita Prefecture Department of Agriculture, Forestry and Fisheries, the rates of rice with a Cd concentration above the safety standard in 2001, 2002, 2003, and 2004 were 13.5, 14.3, 13.3, and 0% in area A, and 37.5–39.7, 11.8–12.5, 0–3.1, and 0–1.4% in area B, respectively [11]. Since these rates are consistent with the results of the present study, except for the missing data points, it is highly plausible that the rate of rice with a Cd concentration above the safety standard before the initiation of the flooding of paddy fields in area B was approximately 30–40%.

Based on these results on Cd concentrations in self–harvested rice, we estimate that oral Cd exposure levels from rice in female farmers may have abruptly decreased from 2002, around the time of the initiation of the flooding of paddy fields, at the same time in the 2 Cd-polluted areas. However, there was a misalignment of the observational timing and period of oral Cd exposure in female farmers between the 2 areas. In area A, we assessed the full period of changes in oral Cd exposure levels from 2001 to 2006, from the high exposure level before the start of the flooding of paddy fields to the lower level. In contrast, in area B, oral Cd exposure levels at the baseline, in 2003 and 2004, are considered to have actually decreased from those before the start of the flooding of paddy fields. In addition, while the reduction in excessive Cd exposure was clearly initiated in 2004 in area B, the date at which excessive Cd exposure levels started to decline was unclear in area A, but was estimated to be in 2004 according to the data from the Akita Prefecture Department of Agriculture, Forestry and Fisheries. Therefore, the actual observational periods of lower Cd exposure levels after the start of the flooding of paddy fields differed between areas A and B: from 2004 to 2006 for 2 years in area A and from 2004 to 2008 for 4 years in area B.

Blood Cd levels are generally considered to reflect recent oral Cd exposure [1]. However, blood Cd levels in female farmers in the present study did not markedly change during the follow-up period despite the reduction in oral Cd exposure from rice. This result may be derived not only from the short observation periods, which were markedly shorter than the biological half–life of Cd (15–30 years), but also from the originally high blood Cd levels, which might rather reflect a substantial internal Cd accumulation, especially in the liver [1]. When blood Cd levels are high, they are not markedly affected by a change in oral Cd intake because the intestinal absorption rate of Cd is very low. Even in the age classes of 34–49 years in areas A and B with the lowest Cd exposure, blood Cd levels were approximately 3.5 and 2.8 μg/L, respectively, which are markedly higher than that in general population in Japan at 1.2 μg/L according to Ikeda [16] or in other countries, such as 0.52 μg/L in the U.S. [17], 1.4 μg/L in England [18] 0.2 μg/L in Sweden [19], 0.74 µg/L in Korea [20], and 0.25 µg/L in Brazil [21]. Therefore, the stable blood Cd levels observed in this follow-up study may be attributed to a constant supply from Cd that has highly accumulated in the liver, which may not be affected by changes in oral Cd intake. A small number of older subjects with extremely high blood Cd levels in area B showed a decrease in the follow-up period. This result may be derived from a decrease in the Cd-holding capacity of the liver due to aging [22] rather than a reduction in oral Cd exposure.

Urinary Cd levels indicate renal Cd concentrations, reflecting the total amount of Cd accumulated in the kidneys due to lifelong exposure. These levels were higher in subjects in area B than in those in area A at baseline; however, during the follow-up period, urinary Cd levels in the elderly classes increased in area A and decreased in area B. A more detailed examination of these changes across all age classes in both areas revealed that urinary Cd levels increased when they were <~5 μg/g cr. at baseline and decreased when they were >~5 μg/g cr. Therefore, a urinary Cd level of 5 μg/g cr. may be an age-relevant balancing point for the accumulation and excretion of Cd in the kidneys. When renal Cd accumulation is below this point, typically at a younger age, Cd, which is largely stored in the liver, moves via the bloodstream to accumulate in the kidneys, leading to elevated urinary Cd levels. In contrast, when Cd exceeds that point and accumulates in the kidneys of individuals >70 years, Cd starts to be excreted from the kidneys, possibly due to a reduced renal capacity to hold Cd, leading to a decrease in urinary Cd levels.

The indicators for renal tubular function, urinary α1MG and ß2MG levels, the latter of which is more sensitive than the former, were higher in subjects ≥70 years at baseline in area B, and continued to increase and show wider differences from those in area A in the follow-up. Renal tubular function deteriorated during the follow-up period in subjects ≥70 years with high baseline urinary Cd levels even though their urinary Cd levels decreased. This contradictory change was more clearly demonstrated in the age-classified and urinary-Cd graded analysis. During the follow-up period, urinary Cd levels increased at lower urinary Cd levels and decreased at higher urinary Cd levels in each age class, indicating a “seesaw relationship” on the axis of urinary Cd levels of ~5 to 7 µg/g cr. On the other hand, urinary ß2MG levels showed an approximately 3-fold increase during the follow-up period in subjects ≥70 years with a baseline urinary Cd level ≥7 µg/g cr. This is consistent with the findings of our previous cross-sectional study in the same Cd-polluted areas in Akita prefecture, where urinary ß2MG levels in subjects aged 70–79 years with urinary Cd levels of 6.5–10 µg/g cr. were higher than those with urinary Cd levels of 3.0–4.5/4.5–6.5 μg/g cr. [9]. Therefore, the urinary Cd level of around ≥7 µg/g cr. would be an empirical risk level to induce renal tubular dysfunction among populations with excessive Cd exposure, under the condition of age >70 years. These results enable us to imagine an initial stage of the sequential development of Cd-induced renal tubular dysfunction in relation to aging. At low levels of Cd exposure, Cd gradually accumulates in the kidneys in proportion to the Cd exposure level, leading to elevated urinary Cd levels without renal tubular dysfunction. After reaching the risk level and surpassing 70 years, the kidneys begin to show damage that leads to the loss of renal Cd into the urine and a subsequent decline in urinary Cd levels, with the concurrent gradual deterioration of renal tubular function.

In addition to the initial stage, which was based on observations of the population, a careful inspection of changes in urinary ß2MG levels in individual older subjects during the follow-up period revealed the final stage in the sequential development of Cd-induced renal tubular dysfunction, namely, the onset of genuine Cd nephropathy, which is diagnosed based on an elevated blood or urinary Cd level and urinary ß2MG level, typically >10,000 µg/g cr. [15]. We identified 3 subjects ≥70 years who were diagnosed with Cd nephropathy during the follow-up period, all of which showed extremely rapid and large elevations in urinary ß2MG levels, up to >20,000 µg/g cr. Urinary ß2MG levels in one subject rapidly increased in only 18 months from 4220 to 23,700 µg/g cr. These results give a characteristic image of the temporal development of Cd nephropathy: among individuals who have been chronically exposed to sufficiently high Cd levels to reach the threshold, a person, often a woman aged ≥70 years, presents with the gradual progression of mild renal tubular dysfunction, and Cd nephropathy suddenly and sporadically occurs, with the acute and rapid progression of renal tubular dysfunction within a very short period.

Previous follow-up studies indicated that after a reduction in excessive environmental Cd exposure, renal tubular dysfunction progressed even though blood or urinary Cd levels decreased, suggesting its irreversibility, often when the urinary ß2MG concentration was >1000 µg/g cr. and the urinary Cd concentration was >10 µg/g cr. [23,24,25] or 8–9 µg/g cr. [26]. Another study reported that urinary ß2MG levels improved after the Cd exposure level was reduced; however, subjects were younger at baseline [27]. These findings are consistent with the present results showing that subjects ≥70 years with a urinary Cd level ≥7 µg/g cr. presented with the deterioration of renal tubular function. The urinary Cd level threshold was originally estimated to be 10 µg/g cr. from the critical level of Cd in the renal cortex required for the appearance of tubular proteinuria [28]; however, detailed analyses in this study, including individual observations of the temporal development of Cd nephropathy, enabled us to derive a slightly lower value.

There may have been limitations to this study that need to be addressed. As noted in our previous report, the subjects in this study were considered to have been chosen with minimal selection bias at the baseline [9]. They lived in areas with the same climate, geographical features, and similar lifestyles. On the other hand, we achieved high follow-up rates, approximately 80% in both areas, but the final results may still be biased due to non-participants, including those lost to follow-up. They might have failed to participate because they had developed or died from diseases related to Cd, leading to an underestimation of the health effects of Cd exposure. We planned to observe changes in Cd accumulation levels in the bodies of female farmers and renal tubular function after the start of the flooding of paddy fields in 2 Cd-polluted areas; however, the timing of the initiation of flooding and the observational periods differed between these areas. Nevertheless, changes in oral Cd exposure levels did not exert any observable effects, suggesting that the results observed in this study were chronological changes under a steady state of excessive internal Cd accumulation rather than the effects of decreases in oral Cd exposure. In addition, the observational periods were short. Even so, we noted distinct changes in renal tubular function as well as renal Cd accumulation levels with age, which provides an outline of the whole picture of the sequential development of Cd nephropathy. We observed the changes in blood and urinary Cd levels during the follow-up period, but it is necessary to address regression to the mean; that is, the extremely high levels in older subjects might have decreased just as a natural variation. Since the extent of decreases was very large (often to less than half), and it has been reported that renal Cd levels decrease over approximately 60 years [1], it is reasonable to consider that the observed decreases in older subjects were a biological phenomenon, not a statistical artifact. After the age or urinary Cd classification, the numbers of subjects who were elderly or had high urinary Cd levels decreased, which sometimes affected the power of statistical tests. However, individual observations enabled us to detect a few genuine cases of Cd nephropathy.

## 5. Conclusions

We conducted a follow-up study on female farmers 4 to 5 years after the flooding of paddy fields was initiated in Cd-polluted areas of Akita prefecture. Cd concentrations in the farmers’ home-harvested rice markedly decreased, indicating that their Cd intake was also reduced. Nevertheless, their blood Cd levels did not change because the reduction in Cd intake may have been too small to affect high blood Cd levels at baseline. On the other hand, elderly farmers with high Cd exposure at baseline showed a deterioration of renal tubular function accompanied by a decrease in urinary Cd levels. Among them, three cases of Cd nephropathy developed, presenting with rapid and large increases in urinary ß2MG levels. Therefore, this study presents a temporal picture of the sequential development of Cd nephropathy.

## Figures and Tables

**Table 1:** Chronological changes in cadmium concentrations in rice (μg/g) in areas A and B.

Area	Area A			Area B		
Cropped year	2001 ^a^	2002	2006	2003	2004	2008
Number	643	114	738 ^b^	240	198	404 ^b^
Median	0.149	0.177	0.089 *	0.156	0.085	0.098 †
(25–75th percentile)	(0.092–0.238)	(0.113–0.275)	(0.048–0.143)	(0.081–0.254)	(0.047–0.154)	(0.056–0.150)
Range ^c^	<0.010–0.971	0.031–0.730	0.006–0.518	<0.020–0.687	0.008–0.669	0.006–0.910

^a^ Newly harvested rice in 2001 (570) and one-year stored rice in 2002 (73). ^b^ One rice sample was not provided. ^c^ The limits of quantitation were 0.010, 0.020, and 0.005 μg/g in 2001, 2002 to 2003, and 2004 to 2008, respectively. * Significant difference from 2001 and 2002 in area A (*p* < 0.05 using the Steel–Dwass test). † Significant difference from 2003 in area B (*p* < 0.05 using the Steel–Dwass test).

**Table 2:** Numbers and mean ages of subjects for analysis.

	Area A		Area B	
	Baseline	Follow-Up	Baseline	Follow-Up
All subjects				
Number	483		295	
Mean age ± SD	57.3 ± 10.1	62.1 ± 10.0 *	57.1 ± 8.3	61.7 ± 8.5 *
Min–Max	22–78	27–83	34–76	40–81
≤33 years				
Number	15		0	
Mean age ± SD	28.6 ± 3.7	33.5 ± 3.6 *	−	−
34–49 years				
Number	78		57	
Mean age ± SD	44.5 ± 3.8	49.3 ± 3.8 *	45.1 ± 3.9	49.6 ± 3.9 *
50–59 years				
Number	167		116	
Mean age ± SD	54.4 ± 2.9	59.3 ± 3.0 *	54.7 ± 2.7	59.1 ± 2.8 *
60–69 years				
Number	187		104 (103)	
Mean age ± SD	64.6 ± 2.8	69.4 ± 2.9 *	63.8 ± 2.7	68.5 ± 3.0 *
≥70 years				
Number	36		18 (16)	
Mean age ± SD	72.4 ± 2.3	76.9 ± 2.4 *	72.1 ± 2.1	77.2 ± 2.5 *

Baseline and follow-up indicate the investigation years: 2001–2002 and 2006 in area A, and 2003–2004 and 2008 in area B, respectively. Age data are presented as the arithmetic mean ± SD. The numbers in parentheses indicate subject numbers that exclude those with missing data on blood and urinary cadmium concentrations. * Significant difference from the baseline (*p* < 0.05 using the Tukey–Kramer test).

**Table 3:** Age-classified cadmium concentrations in peripheral blood (μg/L) in areas A and B (total number = 760).

	Area A		Area B	
	Baseline	Follow-Up	Baseline	Follow-Up
Total	3.72 (2.72–5.17)	3.80 (2.80–5.20)	3.40 (2.30–5.05)	3.50 † (2.50–4.70)
Range	0.55–13.1	0.86–19.0	0.74–16.3	1.00–12.0
34–49 years	3.40 (2.40–5.25)	3.55 (2.68–4.53)	2.78 (1.81–4.53)	2.90 (1.95–4.55)
50–59 years	3.22 (2.42–4.69)	3.60 (2.70–4.80)	3.06 (2.03–4.00)	3.05 (2.33–4.00) †
60–69 years	4.15 (3.06–5.25)	4.10 (3.10–5.40)	3.95 (2.85–6.02) #	4.00 (3.10–5.50) ^a^#
≥70 years	4.51 (3.14–6.04)	4.25 (2.93–6.35)	5.81 (4.24–8.69) #	5.95 (4.03–7.78) ^b^#
J–T test	*p* < 0.001	*p* = 0.003	*p* < 0.001	*p* < 0.001

Baseline and follow-up indicate the investigation years: 2001–2002 and 2006 in area A, and 2003–2004 and 2008 in area B, respectively. Data on cadmium are presented as the median (25–75th percentile). ^a^: one data point is missing. ^b^: two data points are missing. † Significant difference from area A (*p* < 0.05 using the Steel–Dwass test). # Significant difference from the 34–49 years class (*p* < 0.05 using the Steel–Dwass test), J–T test: the Jonckheere–Terpstra trend test.

**Table 4:** Age-classified cadmium concentrations in urine (μg/g cr.) in areas A and B (total number = 760).

	Area A		Area B	
	Baselines	Follow-Up	Baselines	Follow-Up
Total	4.33 (3.01–6.03)	5.37 (3.97–6.94) *	6.06 (4.27–9.00) †	5.43 (4.00–7.53)
Range	0.51–27.3	1.41–17.3	0.61–29.7	1.03–21.5
34–49 years	3.25 (2.36–5.24)	3.75 (2.92–5.77)	3.92 (2.47–5.12)	4.16 (3.39–5.71)
50–59 years	4.05 (2.87–5.91)	4.98 (3.96–6.61) *#	5.87 (4.22–7.57) †#	5.13 (3.84–6.53)
60–69 years	4.81 (3.70–6.87) #	6.11 (4.57–7.37) *#	7.62 (5.78–10.1) †#	6.34 (4.81–8.66) ^a^#
≥70 years	4.09 (3.12–6.07)	6.06 (3.94–7.33) #	9.38 (6.28–10.8) †#	8.65 (6.01–10.5) ^b^#
J–T test	*p* < 0.001	*p* < 0.001	*p* < 0.001	*p* < 0.001

Baseline and follow-up indicate the investigation years: 2001–2002 and 2006 in area A, and 2003–2004 and 2008 in area B, respectively. Data on urinary cadmium are presented as the median (25–75th percentile). ^a^: one data point is missing. ^b^: two data points are missing. * Significant difference from the baseline (*p* < 0.05 using the Steel–Dwass test). † Significant difference from area A (*p* < 0.05 using the Steel–Dwass test). # Significant difference from the 34–49 years class (*p* < 0.05 using the Steel–Dwass test). J–T test: the Jonckheere–Terpstra trend test.

**Table 5:** Age-classified renal tubular function in areas A and B (total number = 763).

	Area A		Area B	
	Baseline	Follow-Up	Baseline	Follow-Up
Urinary α_1_-microglobulin (mg/g cr.)				
Total	4.80 (3.04–8.27)	5.04 (3.19–8.06)	4.32 (2.72–7.15)	5.52 (2.95–8.70)
Range	ND–31.3	ND–46.7	ND–48.6	ND–90.7
34–49 years	2.68 (2.02–5.01)	3.18 (2.29–4.90)	2.80 (2.07–4.43)	2.72 (1.86–5.63)
50–59 years	4.64 (3.10–7.80) #	4.88 (3.40–7.19) #	4.17 (2.53–6.14) #	4.68 (2.96–8.01) #
60–69 years	6.06 (3.86–9.41) #	6.01 (3.66–9.34) #	5.58 (3.55–7.83) #	7.00 (4.06–10.4) #
70 years≤	7.22 (3.58–13.0) #	6.67 (4.82–13.7) #	11.2 (4.33–14.5) #	12.9 (6.51–27.5) #
J–T test	*p* < 0.001	*p* < 0.001	*p* < 0.001	*p* < 0.001
Urinary β_2_-microglobulin (μg/g cr.)				
Total	150 (99–250)	156 (97–260)	150 (96–273)	174 (100–322)
Range	ND–3260	ND–21,200	ND–15,300	ND–33,400
34–49 years	104 (77–154)	103 (72–159)	107 (70–157)	105 (70–196)
50–59 years	147 (96–251) #	154 (108–265) #	144 (88–225) #	161 (99–263) #
60–69 years	166 (116–267) #	174 (105–267) #	174 (116–289) #	227 (129–376) †#
≥70 years	224 (115–401) #	200 (114–642) #	423 (163–884) #	846 (293–2900) †#
J–T test	*p* < 0.001	*p* < 0.001	*p* < 0.001	*p* < 0.001
eGFR (mL/min/1.73 m^2^)				
Total	82.6 ± 14.8	74.8 ± 13.7 *	78.6 ± 14.4 †	79.7 ± 16.6 †
Range	43.5–143.5	26.3–119.2	28.2–133.0	16.9–185.7
34–49 years	88.3 ± 15.1	80.7 ± 12.1 *	84.6 ± 15.6 †	86.8 ± 18.9
50–59 years	84.3 ± 13.3	77.1 ± 11.4 *	79.3 ± 12.4	80.9 ± 13.1
60–69 years	80.6 ± 14.7 #	72.8 ± 13.9 *#	76.8 ± 12.9 #	77.3 ± 15.7 #
≥70 years	72.7 ± 15.2 #	61.3 ± 14.3 *#	65.9 ± 20.9 #	63.3 ± 20.7 #
J–T test	*p* < 0.001	*p* < 0.001	*p* < 0.001	*p* < 0.001

Baseline and follow-up indicate the investigation years: 2001–2002 and 2006 in area A, and 2003–2004 and 2008 in area B, respectively. Data on urinary α_1_-microglobulin and β_2_-microglobulin levels are presented as medians (25–75th percentile), and data on eGFR as the arithmetic mean ± SD. ND: not detected (α_1_-microglobulin, <0.9 mg/L; β_2_-microglobulin, <70 μg/L). * Significant difference from the baseline (*p* < 0.05 using the Steel–Dwass test). † Significant difference from area A (*p* < 0.05 using the Steel–Dwass test). # Significant difference from the 34–49 years class (*p* < 0.05 using the Steel–Dwass test). J–T test: the Jonckheere–Terpstra trend test.

**Table 6:** Chronological changes in blood and urinary cadmium levels and urinary ß_2_-microglobulin levels in a subject who was followed up after the health examination in area A.

	Nov-2002	Nov-2006	May-2008	Nov-2008
Age	66	70	71	72
Blood cadmium (µg/L)	12.90	8.60		14.38
Urinary cadmium (µg/g cr.)	11.88	13.04	12.28	9.29
Urinary ß_2_-microglobulin (µg/g cr.)	1710	4220	23,700	18,000

## Data Availability

The raw data supporting the conclusions of this article will be made available by the authors on request.

## Supplementary Materials

The following supporting information can be downloaded at: https://www.mdpi.com/article/10.3390/toxics14100872/s1. Table S1A: Age-classified urinary creatinine (mg/dL) in areas A and B (total number = 763); Table S1B: Age-classified body weight (kg) in areas A and B (total number = 762); Table S2: Age- and urinary cadmium-classified age, blood and urinary cadmium, and urinary α_1_-microglobulin and ß_2_-microglobulin levels in Area A; Table S3: Age-classified age, blood and urinary cadmium, and urinary α_1_-microglobulin and ß_2_-microglobulin levels in subjects from Area B with urinary Cd < 7 µg/g cr.; Figure S1: Participant flow diagram of the study; Figure S2: Relationships of blood cadmium concentrations between age-classified subjects at the baseline and in the follow-up in areas A and B. Straight lines indicate hypothetical relationships with equal values at the baseline and in the follow-up, and dotted lines indicate actual linear regression lines between the baseline and follow-up; Figure S3A: Individual changes in urinary ß2-microglobulin levels in subjects aged 34–49 years during the follow-up period, presented separately in groups divided by urinary cadmium levels of 3, 5, and ≥7 µg/g cr; Figure S3B: Individual changes in urinary ß2-microglobulin levels in subjects aged 50–59 years during the follow-up period, presented separately in groups divided by urinary cadmium levels of 3, 5, and ≥7 µg/g cr; Figure S3C: Individual changes in urinary ß2-microglobulin levels in subjects aged 60–69 years during the follow-up period, presented separately in groups divided by urinary cadmium levels of 3, 5, and ≥7 µg/g cr.
