# Supplementary material for: A Follow-Up Study After Reduction in an Excessive Intake of Cadmium from Rice on Changes in Blood and Urinary Cadmium Levels and Renal Tubular Function Among Female Farmers in Cadmium-Polluted Areas in Northern Japan
Source: Toxics. 2026 Sep 30;14(10):872. doi: 10.3390/toxics14100872 (PMC13709927; doi:10.3390/toxics14100872)

A follow-up study after reduction of an excessive intake of cadmium from rice on changes in blood and urinary cadmium levels and renal tubular function among female farmers in cadmium-polluted areas in northern Japan

Hyogo Horiguchi [1,2,*], Etsuko Oguma [1,2], Kayoko Miyamotoa [2], Yoko Hosoi [2] and Fujio Kayama [2]

[1] Department of Hygiene, Kitasato University School of Medicine, Sagamihara 252-0374, Japan; oguma@med.kitasato-u.ac.jp
[2] Department of Environmental and Preventive Medicine, School of Medicine, Jichi Medical University, Shimotsuke-Shi 329-0498, Japan; kayokomy@nifty.com (K.M.); yk_hosoi2005@yahoo.co.jp (Y.H.); kayamafujio@gmail.com (F.K.)
* Correspondence: hhyogo@med.kitasato-u.ac.jp

Supplementary table S1A.

Age-classified urinary creatinine (mg/dL) in areas A and B (total number = 763).

	Area A		Area B	
	Baseline	Follow-up	Baseline	Follow-up
Total	62.8 (41.5–89.6)	62.4 (41.7–90.3)	55.2 (37.0–80.2) †	53.5 (36.1–79.8) †
Range	7.6–328	8.5–258	8.5–179	14.4–346
34–49 years	83.7 (43.8–122)	76.1 (46.3–106)	65.1 (43.5–96.9)	61.1 (40.2–91.5)
50–59 years	65.1 (40.4–91.9)	62.2 (39.6–92.2)	54.3 (37.1–81.2)	64.1 (34.9–84.4)
60–69 years	65.1 (40.4–91.9) #	62.2 (39.6–92.2)	54.3 (37.1–81.2) †#	64.1 (34.9–84.4)
≥70 years	62.5 (39.9–90.0)	57.9 (34.2–95.6)	59.6 (40.1–81.0)	54.5 (36.0–69.5)

Data are presented as the median (25–75th percentile).

† Significant difference from the area A ($p < 0.05$ using the Steel–Dwass test).

# Significant difference from the 34–49 years class ($p < 0.05$ using the Steel–Dwass test).

Supplementary table S1B.

Age-classified body weight (kg) in areas A and B (total number = 762).

	Area A		Area B	
	Baseline	Follow-up	Baseline	Follow-up
Total	55.4 ± 8.1	55.2 ± 8.2	56.5 ± 8.2	55.8 ± 8.5
Range	34.7–87.5	35.0–89.2	34.2–88.5	32.2–88.4
34–49 years	57.3 ± 7.9	57.3 ± 8.0	56.6 ± 6.9	56.4 ± 6.6
50–59 years	56.5 ± 8.1	56.4 ± 8.5	58.5 ± 8.4[a]	58.1 ± 8.6[a]
60–69 years	54.2 ± 8.2#	54.0 ± 8.0#	55.5 ± 8.3	54.2 ± 8.4
≥70 years	52.6 ± 7.0#	52.0 ± 7.1#	50.2 ± 6.6#	47.9 ± 7.3#

Data are presented as the arithmetic mean ± SD.

a: one data point is missing.

Significant difference from the 34–49 years class ($p < 0.05$ using the Tukey-Kramer test).

Supplementary table S2.

Age- and urinary cadmium-classified age, blood and urinary cadmium, and urinary α_1-microglobulin and ß$_2$-microglobulin levels in Area A.

Urinary cadmium	<3 µg/g cr.		3–5 µg/g cr.		5–7 µg/g cr.		≥7 µg/g cr.	
	Baseline	Follow–up	Baseline	Follow–up	Baseline	Follow–up	Baseline	Follow–up
Number								
Total	117		163		107		81	
34–49 years	32		24		13		9	
50–59 years	51		51		42		23	
60–69 years	26		74		45		42	
≥70 years	8		14		7		7	
Mean age ± SD								
34–49 years	43.5± 4.2	48.4± 4.3*	44.6± 3.6	49.6± 3.6*	44.6± 3.1	49.3± 3.1*	47.2± 2.3	51.9± 2.1
50–59 years	54.3± 3.2#	59.1± 3.2*#	54.1±2.9#	58.9± 3.1*#	54.8± 2.7#	59.7± 2.7*#	55.0± 2.5#	59.7± 2.5*#
60–69 years	64.4± 3.2#	69.5± 3.2*#	64.4± 2.8#	69.1± 2.8*#	65.2± 2.7#	69.9± 2.9*#	64.4± 2.7#	69.1± 2.7*#
≥70 years	72.9± 2.6#	77.4± 2.7*#	72.9± 2.6#	77.5± 2.8*#	72.1± 2.0#	76.7± 2.1*#	71.0± 1.2#	75.4± 1.3*#
Blood cadmium (µg/L)								
34–49 years	2.99 (1.73–3.96)	2.75 (2.30–3.70)	3.64 (2.48–4.16)	3.50 (2.83–3.98)	5.23 (2.92–6.66)	4.00 (3.25–5.95)	6.75 (4.15–9.54)†	7.30 (5.90–7.70)†
50–59 years	2.81 (2.23–3.92)	3.20 (2.40–4.60)	3.17 (2.41–4.60)	3.60 (2.70–4.40)	3.32 (2.38–4.59)	3.60 (2.65–4.45)	5.57 (3.85–7.40)†	6.20 (4.10–7.10)†
60–69 years	3.33 (2.53–4.38)	2.90 (2.28–4.48)	3.68 (3.05–4.46)	3.60 (2.88–4.90)	4.67 (3.21–5.37)	4.30 (3.40–5.35)†	5.18 (4.40–6.59)†	5.15 (4.10–6.30)†
≥70 years	3.64 (1.83–5.03)	2.70 (1.60–4.20)	3.96 (2.82–5.84)	4.45 (3.13–5.73)	5.09 (4.44–5.65)	4.70 (3.40–5.90)	8.55 (4.08–8.68)	7.60 (3.90–8.60)
Urinary cadmium (µg/g cr.)								
34–49 years	2.20 (1.83–2.52)	3.00 (2.24–3.91)*	3.61 (3.22–4.47)†	3.65 (3.23–5.01)	5.62 (5.24–5.93†	5.18 (3.89–7.28)†	8.63 (7.45–13.1)†	9.58 (7.81–12.7)†
50–59 years	2.50 (1.82–2.84)	3.99 (3.18–5.40)*#	3.93 (3.40–4.34)†#	4.82 (3.87–5.98)*	5.84 (5.37–6.10)†	5.62 (4.47–7.19)†	8.71 (7.58–9.95)†	7.94 (6.37–9.15)†
60–69 years	2.19 (1.57–2.59)	4.20 (3.70–5.49)*#	4.11 (3.61–4.40)†#	5.20 (4.18–6.53)*	5.87 (5.41–6.39)†	6.56 (6.08–7.67)*†	8.42 (7.52–10.0)†	7.51 (6.32–9.06)†
≥70 years	2.27	3.73	3.69	6.02	5.90	6.63	9.72	9.02

	(2.19–2.70)	(2.55–6.36)	(3.48–4.21)†	(3.45–7.25)	(5.30–6.07)†	(5.38–7.29)	(7.16–10.7)†	(5.90–12.9)
Urinary α_1-microglobulin (mg/g cr.)								
34–49 years	2.47<br>(1.97–4.14)	2.91<br>(2.06–4.63)	2.60<br>(2.11–4.75)	3.22<br>(2.82–5.09)	2.92<br>(1.77–5.26)	2.98<br>(2.08–5.57)	4.18<br>(1.90–6.98)	4.35<br>(3.16–6.39)
50–59 years	3.50<br>(2.41–6.48)	4.31<br>(2.65–6.51)	4.46<br>(3.29–6.31)#	4.88<br>(3.02–6.72)	5.48<br>(3.37–9.57)#	5.31<br>(3.51–8.61)#	8.04<br>(4.09–12.4)	5.66<br>(3.79–8.42)
60–69 years	5.91<br>(4.15–9.79)#	4.84<br>(3.46–8.67)#	5.50<br>(3.49–8.37)#	5.48<br>(3.51–8.62)#	6.51<br>(3.99–9.61)#	6.37<br>(3.66–11.2)#	6.58<br>(4.35–9.41)	6.13<br>(3.90–9.74)
≥70 years	6.26<br>(3.01–10.1)	5.07<br>(4.26–6.30)	8.18<br>(3.22–13.2)#	10.4<br>(4.25–12.0)#	9.87<br>(4.66–13.1)#	9.46<br>(6.70–15.8)#	3.73<br>(3.19–21.2)	6.37<br>(4.63–16.7)
Urinary β_2-microglobulin (µg/g cr.)								
34–49 years	100<br>(82–145)	102<br>(75–157)	104<br>(76–139)	95<br>(59–187)	102<br>(47–204)	111<br>(67–151)	146<br>(99–290)	113<br>(89–239)
50–59 years	150<br>(95–204)	146<br>(114–297)#	133<br>(91–177)	136<br>(99–196)	144<br>(100–283)	177<br>(118–278)	251<br>(110–399)	166<br>(106–335)
60–69 years	164<br>(119–353)#	132<br>(85–207)	153<br>(107–237)#	164<br>(92–266)	186<br>(126–278)#	176<br>(119–311)	175<br>(98–346)	185<br>(141–277)
≥70 years	267<br>(118–364)	156<br>(107–235)	187<br>(90–593)	149<br>(100–561)	306<br>(181–466)	396<br>(293–913)#	184<br>(117–973)	258<br>(172–964)

Data on age are presented as the arithmetic mean ± SD, and others are shown as the median (25–75th percentile).

* Significant difference from the baseline ($p < 0.05$ using the Tukey-Kramer test or the Steel–Dwass test).

† Significant difference from the group with a urinary Cd level <3 µg/g cr. ($p < 0.05$ using the Steel–Dwass test).

# Significant difference from the 34–49 years class ($p < 0.05$ using the Tukey-Kramer test or Steel–Dwass test).

Supplementary table S3.

Age-classified age, blood and urinary cadmium, and urinary α_1-microglobulin and β_2-microglobulin levels in subjects from Area B with urinary Cd < 7 μg/g cr..

	Baseline	Follow–up
Number		
Total	119 (116)	
34–49 years	9	
50–59 years	35	
60–69 years	61(60)	
≥70 years	14 (12)	
Mean age ± SD		
34–49 years	45.3±5.0	50.0±4.6*#
50–59 years	55.3±2.6#	59.8±2.8*#
60–69 years	63.6±2.7#	68.4±2.9*#
≥70 years	72.3±2.3#	77.5±2.4*#
Blood cadmium (μg/L)		
34–49 years	5.93 (4.74–7.87)	5.30 (4.15–7.00)
50–59 years	3.93 (3.05–6.96)	3.80 (3.10–4.60)
60–69 years	4.96 (3.82–7.59)	4.90 (3.90–6.00)
≥70 years	6.34 (5.30–8.96)	6.65 (4.75–8.33)
Urinary cadmium (μg/g cr.)		
34–49 years	9.55 (8.15–11.5)	8.51 (7.33–12.1)
50–59 years	9.08 (7.80–10.5)	7.13 (6.42–8.95)
60–69 years	9.45 (8.52–11.9)	7.93 (6.45–10.8)*
≥70 years	9.85 (9.00–13.4)	10.0 (7.10–10.8)
Urinary α_1-microglobulin (mg/g cr.)		
34–49 years	2.60 (1.67–3.75)	2.68 (1.86–4.69)
50–59 years	4.63 (3.17–6.56)	5.09 (3.45–8.82)#
60–69 years	5.93 (3.57–8.58)#	6.90 (4.07–10.5)#
≥70 years	11.15 (6.10–13.1)#	12.9 (6.51–27.5)#
Urinary β_2-microglobulin (μg/g cr.)		
34–49 years	92 (56–228)	120 (70–176)
50–59 years	191 (119–283)	178 (138–330)

60–69 years	199 (131–337)	236 (127–428)
≥70 years	387 (162–884)#	814 (286–2900)#

Data on age are presented as the arithmetic mean ± SD, and others are shown as the median (25–75th percentile). The numbers in parentheses indicate subject numbers that exclude those with missing data on blood and urinary cadmium concentrations.

* Significant difference from the baseline ($p < 0.05$ using the Tukey-Kramer test or the Steel–Dwass test).

# Significant difference from the 34–49 years class ($p < 0.05$ using the Tukey-Kramer test or Steel–Dwass test).

Supplementary figure S1.

Participant flow diagram of the study.

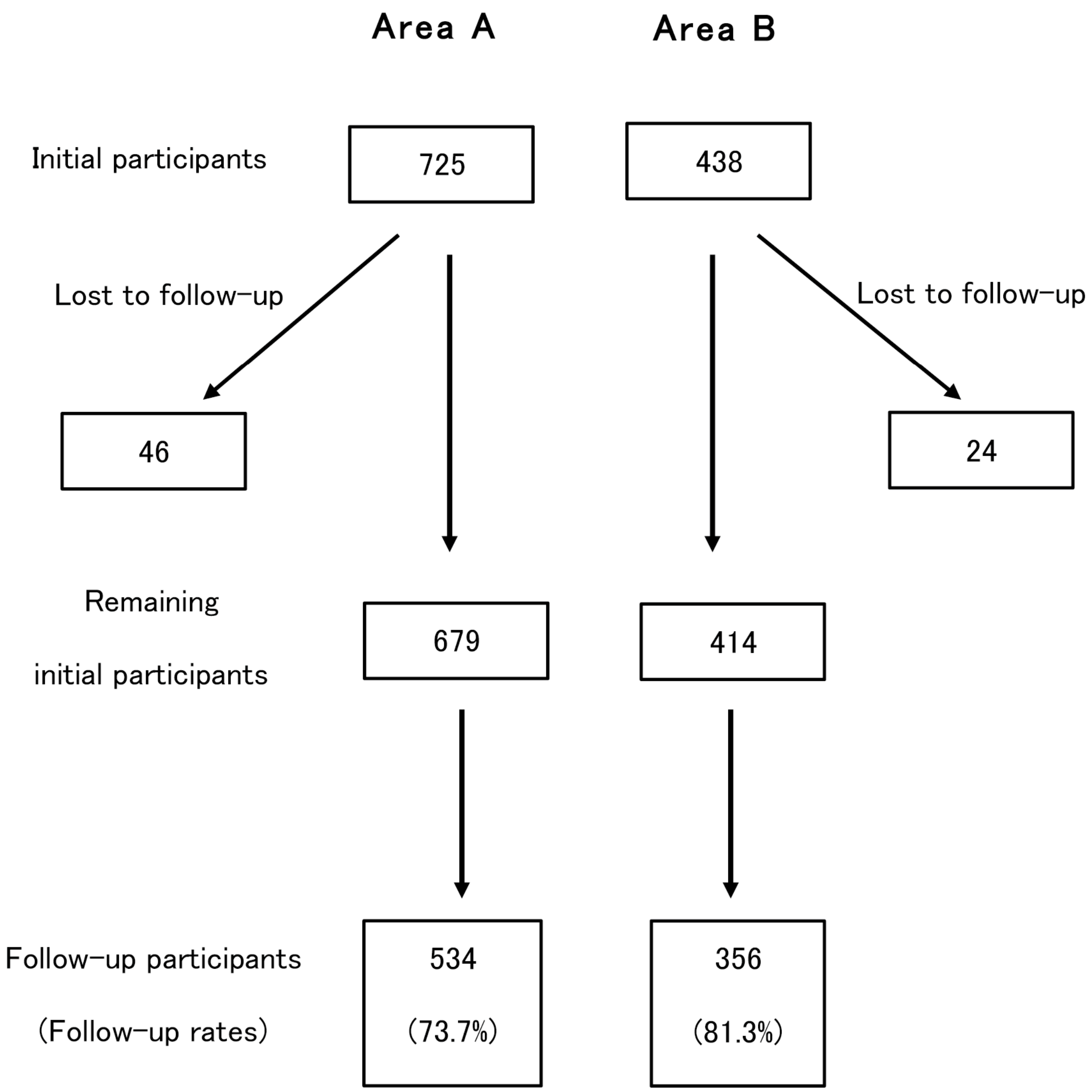

Supplementary figure S2.

Relationships of blood cadmium concentrations between age-classified subjects at the baseline and in the follow-up in areas A and B. Straight lines indicate hypothetical relationships with equal values at the baseline and in the follow-up, and dotted lines indicate actual linear regression lines between the baseline and follow-up.

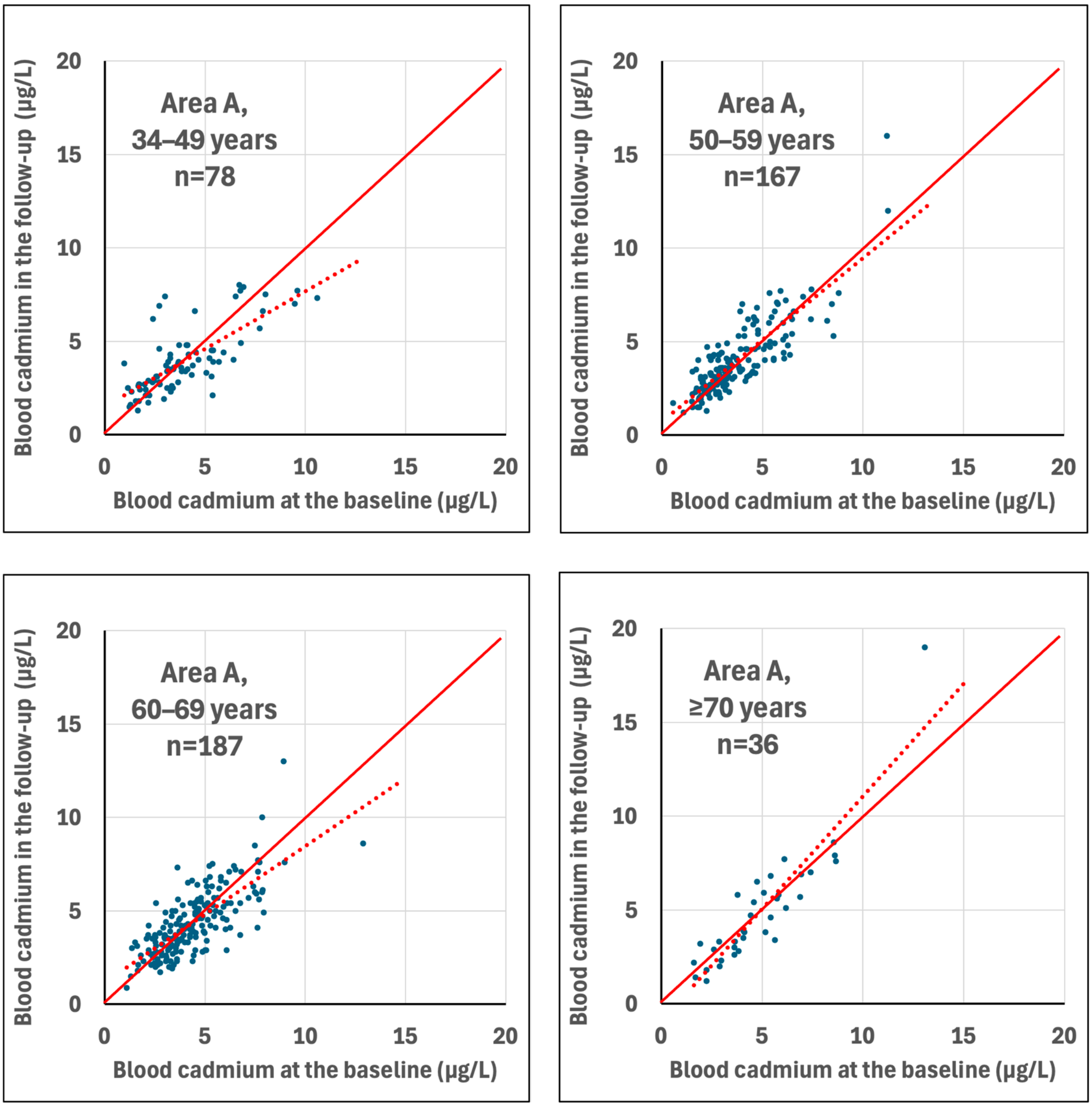

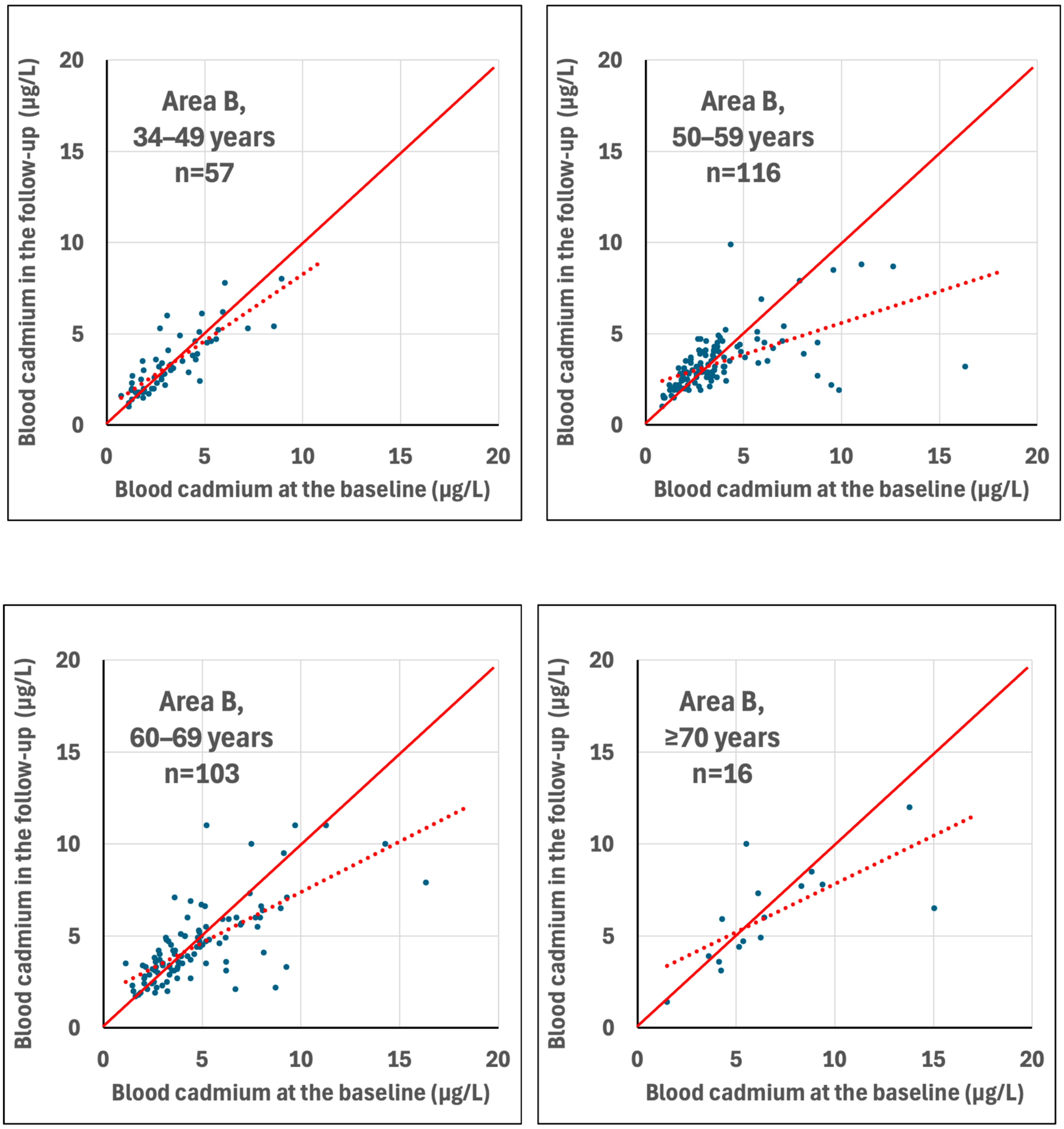
Area B,
34–49 years
n=57
Area B,
50–59 years
n=116
Area B,
60–69 years
n=103
Area B,
≥70 years
n=16
Blood cadmium in the follow-up (μg/L)
Blood cadmium at the baseline (μg/L)
0
5
10
15
20

Supplementary figure S3A.

Individual changes in urinary ß₂-microglobulin levels in subjects aged 34–49 years during the follow-up period, presented separately in groups divided by urinary cadmium levels of 3, 5, and ≥7 μg/g cr.

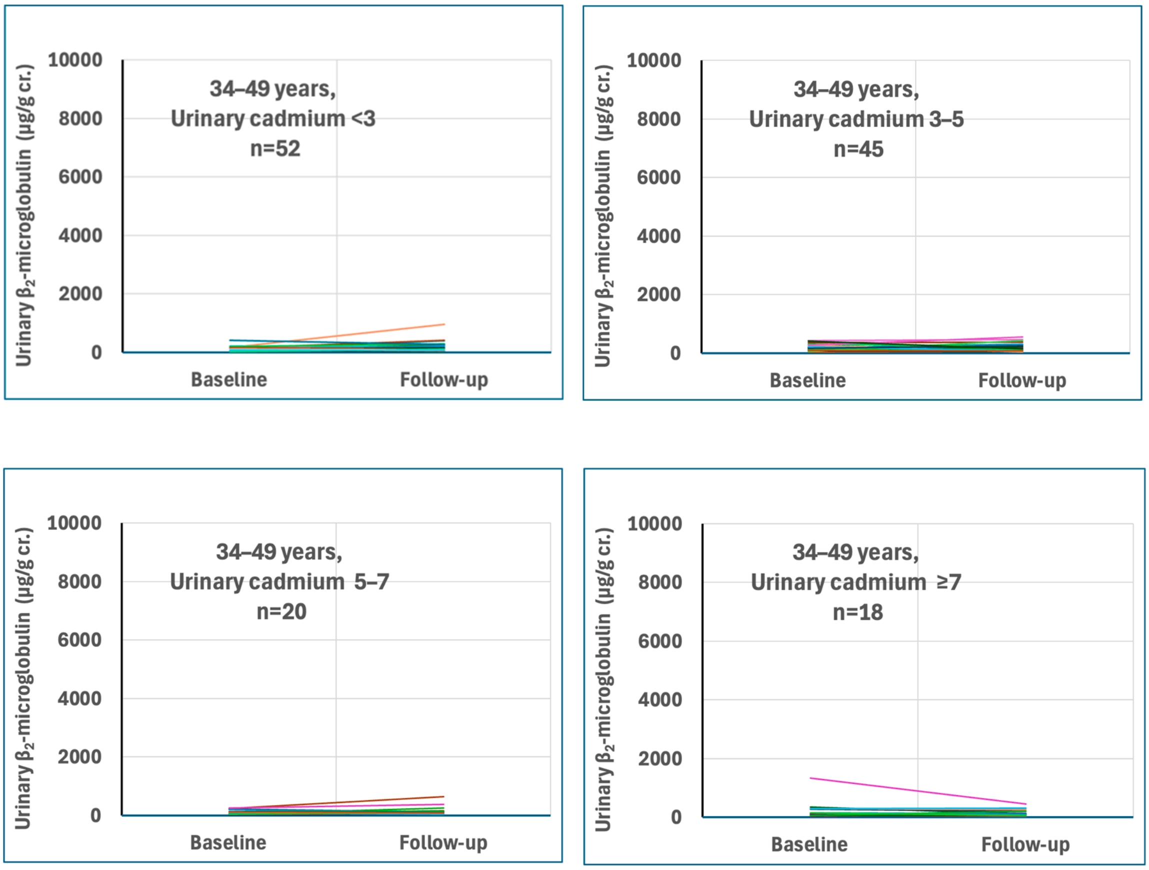

Supplementary figure S3B

Individual changes in urinary β_2-microglobulin levels in subjects aged 50–59 years during the follow-up period, presented separately in groups divided by urinary cadmium levels of 3, 5, and ≥7 μg/g cr.

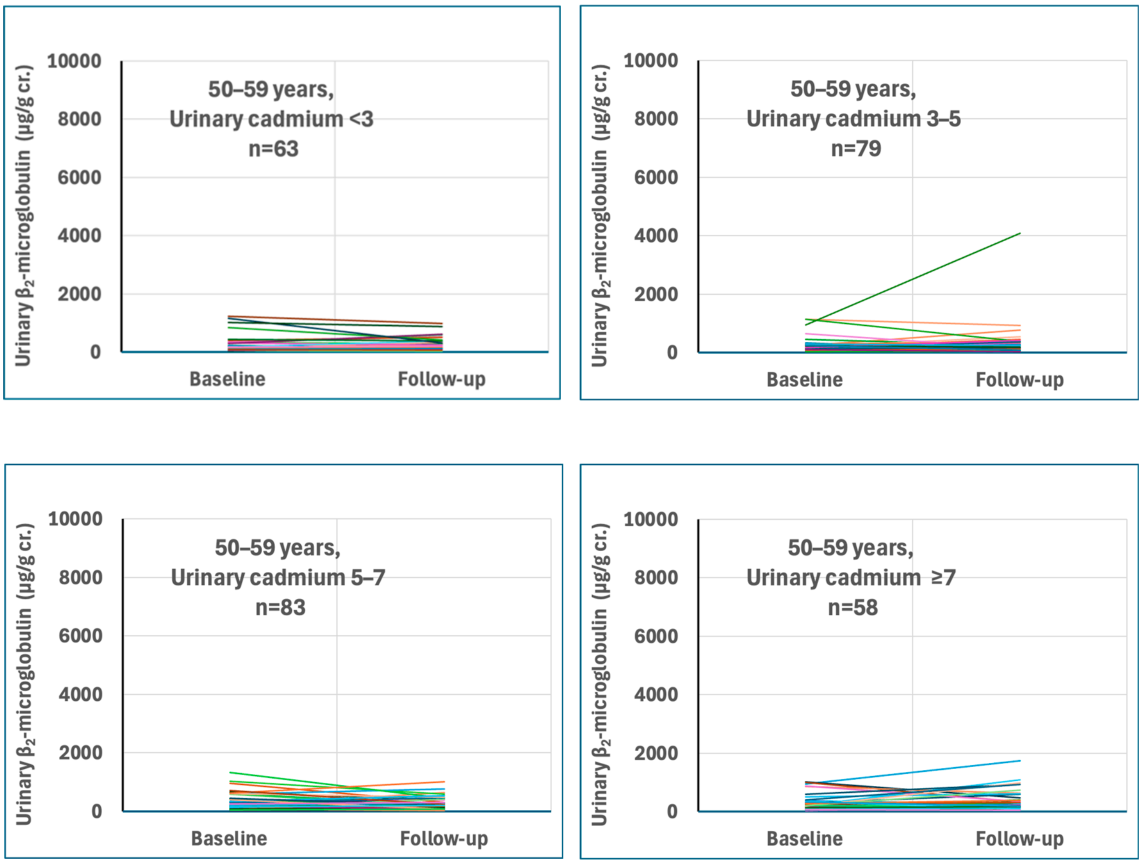

Supplementary figure S3C

Individual changes in urinary β_2-microglobulin levels in subjects aged 60–69 years during the follow-up period, presented separately in groups divided by urinary cadmium levels of 3, 5, and ≥7 μg/g cr.

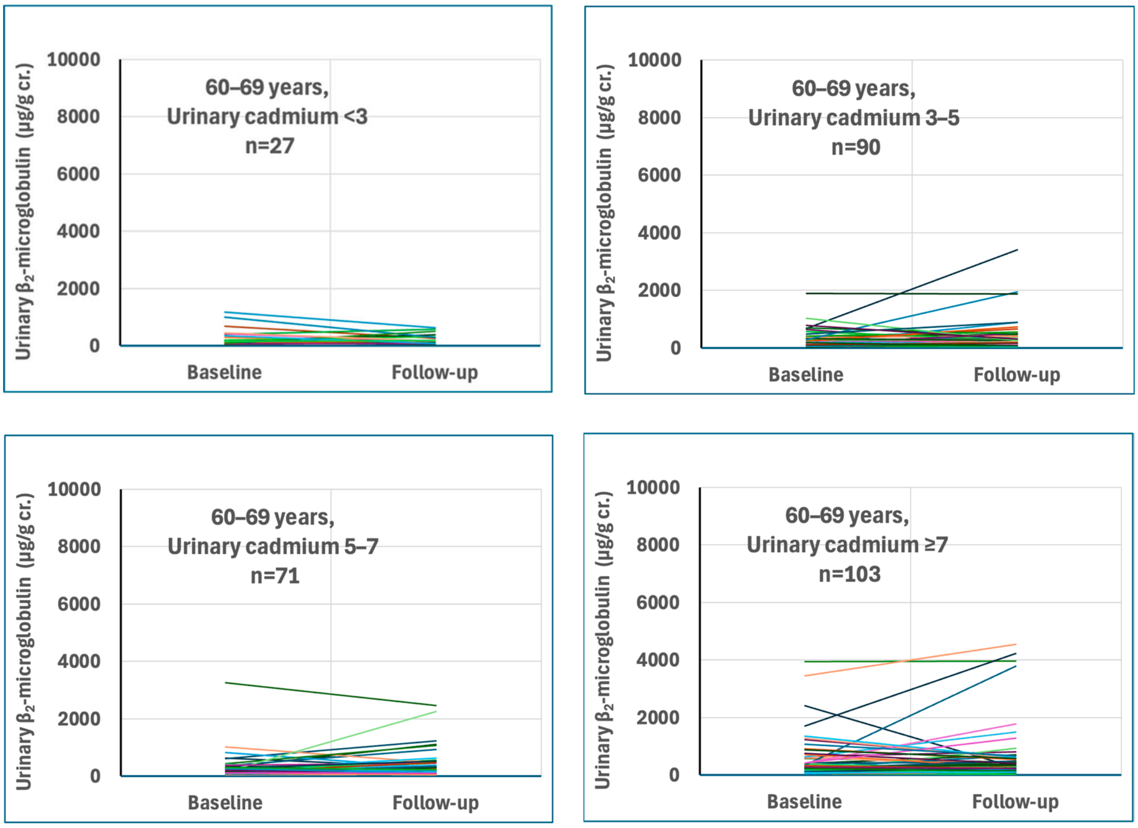

Supplement: Supplementary file 1 [file toxics-14-00872-s001.zip › toxics-4580855-supplementary.pdf]
